# Perspectives on mental health services for medical students at a Ugandan medical school

**DOI:** 10.1186/s12909-022-03815-8

**Published:** 2022-10-25

**Authors:** Raymond Bernard Kihumuro, Mark Mohan Kaggwa, Rachael Mukisa Nakandi, Timothy Mwanje Kintu, David Richard Muwanga, David Jolly Muganzi, Pius Atwau, Innocent Ayesiga, Anita Acai, Sarah Maria Najjuka, Josephine Nambi Najjuma, Susan Frazier-Koussai, Scholastic Ashaba, Sheila Harms

**Affiliations:** 1 Faculty of Medicine, Mbarara University of Science and Technology, Mbarara, Uganda; 2 African Centre for Suicide Prevention and Research, Mbarara, Uganda; 3 Department of Psychiatry and Behavioural Neurosciences, McMaster University, Hamilton, Canada; 4 College of Health Sciences, Makerere University, Kampala, Uganda; 5 Texas Juvenile Crime Prevention Center at Prairie View, A and M University (PVAMU), Texas, USA

**Keywords:** Mental health services, Medical students, Curriculum, Trauma, Stigma, Psychological distress, Uganda

## Abstract

**Background:**

University-based mental health services for medical students remain a challenge, particularly in low-income countries, due to poor service availability. Prior studies have explored the availability of mental health services in high-income countries but little is known about mental health services in countries in sub-Saharan Africa, such as Uganda. Medical students are at a higher risk of developing mental health challenges during their course of study as compared with other students. Thus, there is a need for well-structured mental health services for this group of students. The aim of this study was to explore perspectives on mental health services for medical students at a public University in Uganda.

**Methods:**

This was a qualitative study where key informant interviews were conducted among purposively selected university administrators (*n* = 4), student leaders (*n* = 4), and mental health employees of the university (*n* = 3), three groups responsible for the mental well-being of medical students at a public university in Uganda. Interviews were audio-recorded, transcribed, and thematically analyzed to identify relevant themes.

**Results:**

The working experience of university administrators and mental health providers was between eight months to 20 years, while student leaders had studied at the university for over four years. We identified five broad themes: (1) Burden of medical school: A curriculum of trauma, (2) Negative coping mechanisms and the problem of blame, (3) The promise of services: Mixed Messages, (4) A broken mental health system for students, and (5) Barriers to mental health services.

**Conclusion:**

Distinguishing between psychological distress that is anticipated because of the subject matter in learning medicine and identifying those students that are suffering from untreated psychiatric disorders is an important conceptual task for universities. This can be done through offering education about mental health and well-being for administrators, giving arm’s length support for students, and a proactive, not reactive, approach to mental health. There is also a need to redesign the medical curriculum to change the medical education culture through pedagogical considerations of how trauma informs the learning and the mental health of students.

**Supplementary Information:**

The online version contains supplementary material available at 10.1186/s12909-022-03815-8.

## Introduction

Medical students are at an increased risk of developing psychological distress and mental health disorders relative to other undergraduate students [1, 2]. This vulnerability is hypothesized to be secondary to exposure to a stressful curriculum, which places significant demands on the student in terms of the volume of work, the inherent complexity of learning the practice of patient care, feeling a lack of control over the educational experience, and exposure to death and dying for the first time [1–3]. Robust empirical data from meta-analyses suggests that over one in ten medical students experience mental health stressors that meet diagnostic criteria for psychiatric disorders and related complications. For example, prevalence rates of depression (27.2%), anxiety (33.8%), burnout (12.1%), and suicide (11.1%) for medical students are alarming [4–6]. Although the student populations evaluated in these studies primarily represent high-income settings, low- and middle-income countries (LMICs) have also reported a high pooled prevalence of mental stressors such as burnout, depression, and stress among medical students [4, 7]. Symptoms of depression have been found to be higher among African medical students as compared with medical students from other LMICs [7]. This higher prevalence rate among African medical students has been attributed, in part, to the overwhelming burden of poverty within public systems of learning, which inevitably affect the learning environment [8]. Additional challenging socio-political phenomena in Africa including chronic civil unrest, warfare, low rates of employment, and systemic human rights abuses, such as gender-based violence, also directly influence medical education in addition to the under-resourced healthcare system [9]. In Uganda, more than half (57.4%) of medical students at one university reported being extremely stressed [10], whereas one in five medical students was depressed [11]. At another Ugandan university, 54.5% of the medical students experienced serious burnout [12] and two medical students died by suicide within an 11-year period (2010-2020) [13]. The prevalence of mental health symptoms (depression, anxiety, and stress) was even higher during the COVID-19 pandemic, with over four-fifths of students having severe symptoms [14].

Compromised mental well-being for medical students is associated with student dropout, poor academic performance, substance experimentation and abuse, compromised patient care, and weakened social support systems [1, 2]. In response, medical bodies and associations have increasingly advocated for the improvement of medical students’ mental well-being. For example, the Association of American Medical Colleges has recommended access to confidential counselling by mental health professionals for all medical students, as well as establishing policies for student wellness evaluation, treatment, and confidentiality [15]. However, lack of resources, and in particular, human resources, often keep medical schools and universities from effectively implementing these recommendations [8, 15]. This is especially true for low-income settings such as Uganda. Realities such as these often force universities to rely on a combination of sparsely staffed university counselling centres, community mental health providers, and university faculty who may be called on to provide psychiatric consultation in urgent cases [1, 15]. There are obvious challenges with this approach, including wait times for students, lack of confidentiality, and stigma [15]. However, medical students may benefit from mental health services offered by the university and medical school, particularly through their direct exposure to the mental health providers during their psychiatry clinical rotations (fourth year in Uganda) who teach them how to identify and manage mental health challenges, thereby leading to better self-care [1, 16].

Despite the challenges present in providing mental health services to students, general patterns in the literature suggest that the global use of the available mental health services by medical students is poor, which is mainly attributed to perceived stigma, lack of knowledge about existing services, and students’ poor attitudes towards the offered mental health services [17–20]. A review in Nigeria also found stigma to hinder the mental health-seeking behaviors of medical students [21]. Ugandan literature has recently described an introduction of peer counselling services for medical students at Makerere University in 2002, although this program has not yet been evaluated [22]. No literature exists describing perspectives towards the mental health services currently offered to medical students in Uganda. Therefore, we sought to understand different stakeholders' (i.e., university administrators, student leaders, and mental health employees) perspectives about the mental health services offered to medical students at a large Ugandan university.

## Methods

### Study design and study population

This was a qualitative descriptive study [23] that involved key informant interviews with purposively selected mental health stakeholders in Mbarara University of Science and Technology (MUST). The stakeholders selected included university administrators in the Office of the Dean of Students (i.e., those responsible for students welfare),  student leaders (i.e., those responsible for coordinating activities related to student wellness); staff in the Office of the Dean of the Faculty of Medicine (i.e., those responsible for the academic affairs of students), and mental health service providers (i.e., university counsellors, psychiatrists, and nearby mental health facility health workers) at MUST. This study was reported in accordance with the COnsolidated criteria for REporting Qualitative research (COREQ) checklist [24].

### Study area

MUST is public university in Southwestern Uganda and it is the second largest, and oldest among the 12 universities training medical doctors in the country. MUST is located in Mbarara City, 260 km from Kampala, the capital city. The Faculty of Medicine has 28 departments that offer Bachelor degrees (medicine, surgery, nursing, medical laboratory science, pharmacy and physiotherapy), Master’s degrees (medicine, nursing, public health, and different fields of science), and PhDs (different fields of science). Annually, the Faculty of Medicine graduates approximately 90 doctors per year. As of January 2022, the MUST medical school curriculum runs for five years with students spending two years in pre-clinical training and three in clinical training at the Mbarara Regional Referral and Teaching Hospital. The medical students are exposed to psychiatry in the first semester of their fourth year.

### Data collection

Interviews were conducted by members of the research team trained in qualitative data collection methods (RBK, RNM, DMR, TMK, DJM, PA, and IA). Each interview was led by one member in the presence of at least two members of the team, so that their different memos summarising the interaction were recorded. Interviews took place between July 8, 2020 and July 31, 2021. The interviews were scheduled based on participants’ availability and lasted between 30 to 60 min (average: 40 min). Two informants were interviewed in person (on campus, in the office of the participants) and 9 were interviewed virtually via Zoom due to pandemic restrictions. Participants were purposively selected based on their position in the university. A total of four administrators did not participate in the study due to having a conflict of interest (i.e., helped in the conceptualisation of the study) or some having no time to participate. One mental health provider was not included due to being extremely physically unwell during the period of data collection. However, all student leaders from medical school participated in the study. All interviews were conducted in the English language given that medical education is taught in English and all participants were fluent in English. Interviews were audio-recorded and transcribed by two independent members of the research team (RNM, DMR, TMK, DJM, PA, NJ, SMN, or IA).

The interview guide was generated through reading literature relevant to mental health services among university students [1, 17–20, 25–27] and with input from mental health and medical education experts in the region. The questions from the semi-structured interview guide (Supplementary Material 1) explored the services offered at the university, aspects of their use of mental health services, and future prospects to improve use of the services.

### Analysis

The transcribed documents were checked by RBK for any inconsistencies and the final version was proofread by MMK. Personal identification data were removed at this stage before further analysis. Two of the approved the transcribed interviews were sent to the participants to confirm the accuracy of the transcribed interview and the transcription process. Analysis occurred concurrently with data collection process using thematic analysis [28] to understand and explore perspectives and experiences with the mental health services offered at the university. The specific six steps followed in the thematic analysis [28] were: (i) familiarising oneself with the data, (ii) generating initial codes, (iii) searching for themes, (iv) reviewing themes, (v) defining and naming themes, and (vi) producing the report. After reading three randomly chosen transcripts (one from each of the different stakeholder groups), the research team developed a codebook to guide the analysis for the remaining interviews through relational content analysis. During the analytic process, members of the research team (MMK, RBK, SMN, AA, AS, and SH) held weekly meetings to harmonise the identified themes and to discuss their interpretations of the data. The team reflected a collaborative effort between researchers from Mbarara University (RBK, RNM, DMR, TMK, DJM, PA, IA, MMK, NJ, and AS), and other medical training institutions in Uganda (SMN) and beyond (FK, AA, and SH). This diversity of the research team characteristics helped inform a more robust interpretation of the data. As data analysis was ongoing, we kept collecting data until data sufficiency was achieved. After analysis, we conducted member checking to ensure trustworthiness of the data [29]. The summaries of the analysis were read out to two of the participants to check if the interpretation of the information was aligned with their experiences, to which they concurred.

### Ethics

The study received ethical review and approval from the Research Ethics Committee of MUST (MUST-2021-99). Permission to collect data from participants was granted by the Dean of Students at MUST. All participants provided voluntary, written informed consent before the interviews were conducted. Potential participants were contacted via email and given a copy of the informed consent form as well as a link to a Google Form containing the detailed consent form and an option to schedule an interview if they were interested and consented to participate. At the start of data collection, the consent form was reviewed with each participant to make the information clear to the participants. Participants who had their interviews via Zoom consented to have the interviews recorded.

## Results

### Participant demographics

Eleven key informant interviews were conducted (i.e., five females and six males, age range of 22 to 60 years). The university staff had a working experience with the university of between eight months to 20 years, serving in various positions including university administrators (*n* = 4) and mental health employees of the university (*n* = 3). The student leaders (*n* = 4) had studied for four to five years at the university. Three of the administrators were also physicians and one was a psychiatrist.

#### Theme 1: Burden of medical school: A curriculum of trauma

Students spoke about the lack of knowledge about what medical school and practice of medicine entailed prior to applying for the course. For medical students, the learning experience was stressful at every stage of training, including experiencing a kind of “buyer’s remorse” in the sense that learners did not know what they were getting themselves into prior to studying medicine. Students spoke about never being told the demand of the course before joining but only the advantages of being a doctor. One student said,

*“Unfortunately, when we are being guided career-wise [before medical school], we are really never told what exactly it entails. So, when we get to medical school it gets hard.”* (Interview 9, Student Leader)

Students also described a lack of mentorship from people who had gone through the medicine course, which did not prepare them for emotional agonies such as witnessing someone die. Furthermore, students recognized that these learning encounters with serious illness and death were not being discussed or processed as part of their curriculum while at the same time recognizing that the existing structures of “peer support” meant that they were responsible for taking care of each other’s mental well-being in response to these traumatic events. Students acknowledged that their own mental health was being affected by the curriculum; however, the demands of the training program were described as extremely intensive and time-consuming, such that when students experienced mental health symptoms, they had no time to go for help or assistance. The issue of lack of time was particularly salient for most of the student leaders and university mental health providers, as illustrated by this comment:

“*The medical students … [are] busy most of the time, … so, for that reason, you find that getting them is a challenge. Counselling is a process; it is not a one-day thing. If the first time I have done a rapport, then the next time I want to start the counselling. Now, the student stops at building the rapport. The next day he is not able to come back because of the nature of his course and that also hinders the counselling process.”* (Interview 7, University Counsellor)

All participants, irrespective of their position in the university, described the medical school experience as extremely difficult. At times, learning was described as a traumatising experience where students unexpectedly underwent learning encounters never previously anticipated or discussed.

*“The course content is hard, what it takes is hard; the ward rotations, the interactions with doctors, the meeting of death, the first time you lose a patient, the first time you see breath leave someone, those things can get depressing. And also, the stress itself, I mean you are around sick people all the time.”* (Interview 9, Student Leader)

The burdens in medical school curriculum were also emphasized by one of the faculty of medicine administrators who said:

“*Medical school is the most stressful course. Training as a doctor is the most stressful course on earth. You find that medical students are the ones who are most stressed. The curriculum is so tight, you are studying from eight or even seven. You go to the ward. In the ward there are many challenges, you are going to see sick people, you see, going to see people who are dying. And then there is lots of work to read. Some courses are tough and so medical school is very stressing.*” (Interview 11, Administrator)

#### Theme 2: Negative coping mechanisms and the problem of blame

Due to the demands and perceived stressors associated with the training program, many students described turning to the use of drugs and alcohol to cope. Students described a feeling of impending breakdown and struggled not only within the medical school program, but also as an effect of the training that impacted their personal lives, such as romantic relationships. The feeling of being overwhelmed and helpless can be read from the following quote, where the student clearly linked affective struggle with the use of substances:

*“The toll of pressure was a lot and some people resorted to drugs and all that, and I had my own tolls of overweight, mainly academic but also from outside relationships and all those pressures that come from academics, from dating, you always feel you are overwhelmed, you always feel like you are iminent... for a breakdown but you have no access for help … or you don’t know where to access for help.”* (Interview 9, Student Leader)

*Some of them tell me your students [medical students], some use drugs. That is why they are having break downs. … They engage in risky sexual behaviours, don’t want to study, and they don’t realize that some of these issues are now really imminent.”* (Interview 8, Student Leader)

Administrators, mental health counsellors, and students recognized that students were struggling to cope but ultimately saw the use of drugs as dangerous, risky, and illegal. The use of addictive substances was considered the main cause of student mental health challenges by most administrators in the university. Most participants did not acknowledge the role of substances in student coping. Administrators mentioned that in Uganda, the use of drugs is illegal, which therefore led to a discourse of criminalization that superseded concerns about student mental health. Students were blamed for using such methods to cope with the stress of the program or to self-medicate emerging psychiatric symptoms. The criminalization of substance use was clearly articulated by the students as being a deterrent in seeking mental health services. The following comment illustrated how students coped as well as their attitudes about being considered criminal in their attempts to self-medicate:

*“Most of the students that I have encountered that have mental health issues actually have bad coping mechanisms. So, some resort to either drugs others resort to unexplained activities or indiscriminate sexual activity or certain things like that and it really hurts me that very many people in the university instead of facing their mental health issues resulting from their social and academic lives. They tend to resort to coping mechanisms that are not sustainable and may not stand the test of time.”* (Interview 8, Student Leader)

*“The first thing, most especially like now for substance abuse being a crime, most students really fear to get help from the university. Not knowing that by virtue of being a university student, as long as you go through the university process, there’s a certain form of protection that the university will try to give you until they absolutely can’t. …. So, the majority of the students for those who are engaged in criminal activity will fear to actually getting help from the university.”* (Interview 5, Student Leader)

#### Theme 3. The promise of services: Mixed messages

Mental health services were introduced to the students during their first year in university through an orientation process. However, the clarity of the messages was compromised and instead, resulted in mixed messages between the different stakeholders. Almost all administrators and counsellors believed that the message of availability of mental health services was clearly delivered during first year study orientation, including information about the process of accessing services. However, students claimed that few were in attendance during the orientation and that vital information was not communicated. Opportunities to hear about mental health services were not available after the first year. One administrator said:

“*Usually, they get to know about them when they are reporting to campus during orientation. I know that the Office of Dean Students usually speaks about the services available to students. When we have some specialists invited to give some brief talks, they hint about the availability of some of these services. I know that students when they are rotating through the Department of Psychiatry (in the fourth year of medical school), they get to know about the services offered there. Another way that students get to know is a peer. I know of students who have said they got to know about services from their colleagues.”* (Interview 4, Administrator)

*“When the students come into the university in the first year … all of them are oriented, they must attend an orientation process where different personnel in the university, different relevant people in the university come and explain to them the different services offered. I realised while that service is offered, the attendance is quite low, most first years do not attend that program.”* (Interview 5, Student Leader)

Administrators added that the specific mental health services offered by the university included several different options such as peer support systems, counselling services, and psychiatric treatment offered at the psychiatry hospital. The perspectives about the availability and effectiveness of the described services varied widely among the stakeholders.

##### Administrator confidence and conviction

The administrators were very confident in the services they offered, emphasising the ways in which services had been advertised and promoted, such as advertising through university websites, encouraging students to take advantage of the well-established peer support system, relying on student leaders to help triage mental health needs within the student population, and accessing the two active university counsellors. However, students believed otherwise. Administrators made statements suggesting that their input was the extent of their responsibility because students were not accessing the offered services. This is clearly described by the following quotation:

“*Of course, the services have been there. I don’t know how else you want us to make them known because they are there. If people can come for the service, we are there to help. … We can’t say we have failed, though the services are not being accessed. For me who offers the service, I am there. It is a known service, like I said when we do orientation for first-year students who are thinking about other things that are going on in their minds and say they have never seen you. You realise they are not there mentally when you are doing orientation. I really do not know how best we can improve the information.”* (Interview 3, Administrator)

Students believed that online platforms could be used to improve the spread of information about the offered mental health services. However, despite the conviction of the administrators and the counsellors, even student leaders reported having little knowledge about services offered or the presence of counsellors. The following quote illustrates this:

*“No, there’s no mental health service that I know of. But I think they [university administration] also realise that there’s a gap. But I think they are not in a rush or they don’t think it is an urgent matter that needs to be handled now because I have tried to interact with them.”* (Interview 8, Student Leader)

Some students only found out about counsellors and the services they offered in their final years of the medical degree program (years four and five):

*“They’ll probably struggle not because that help is not there but because they don’t know that mental health help is there, so the recommendation is that information is spread. I mean we have social media platforms; we have notice boards and engagements. … We have a counsellor, the first time I got to hear of it, I was even shocked, of course, it’s not that I didn’t expect that there is one but the fact that am four years in the university, I don’t even know a friend or a friend of a friend who went to a counsellor.”* (Interview 9, Student Leader)

##### Student leaders are mental health services chaperones

Students, especially student leaders, were trained by the university to lead a peer support system within the university. This involved communicating the available mental health services to other students and helping those with mental health concerns by offering basic counselling or referring them to the university counsellors or administrators. However, students found this to be draining and at times traumatising due to the volume of students who were seen to have mental health challenges. They believed that the number of mental health issues were excessive for student leaders to manage and that the university counsellors were not doing enough to address the issues. A student leader clearly elaborates their perspective in the following comment:

“*Being a leader in the university is quite traumatic; for the leaders themselves because you’re constantly being exposed to the horrors that different people are going through and therefore it is hard to keep a straight face; it is hard to alienate yourself from these experiences and it even takes you back to your own past, your own history, your own family and relatives. It really a tough thing to handle but you have to as a leader, you literally don’t really have a choice. So yes, there are definitely countless mental health issues in the university and around the university that are not being dealt with. I believe the services that we have at the university include having counsellors, we only have one official counsellor, and then the other people are basically posing as counsellors.*” (Interview 5, Student Leader)

##### University mental health counsellors struggle to provide services

The mental health counsellors described trying to do their best to use the available resources (i.e.., limited funding, space, human resources, and support from administration) to provide care to all university students and faculty. However, they described being understaffed and given too many additional responsibilities such as teaching, administrative roles, training peer support teams in the university, and being expected to manage clinical practices within the hospital. The available counsellors whose primary role was to support students were described as being inadequate to meet student needs. The workload was so excessive that counsellors described losing track of what was happening. The conundrum is illustrated by the following quotation:

“*We have about five counsellors who are counselling psychologists by profession, and [they are in different areas of the university]*. *We handle them under the Dean of the Student's Office. But then we are also those who could be referred because of the mental health challenges to the psychiatric department for further management and we know there is a need that a counsellor is also needed on that side.*” (Interview 7, University Counsellor)

#### Theme 4. A broken mental health support system for students

All participants acknowledged that the university system for supporting student mental health was flawed and required repair. One administrator said:

“*We need to identify enough numbers of people who can offer the service. We need to have adequate space where the services can be accessed. We need to involve the students themselves in coming up with possible solutions to challenges that affect them. We need to have a program for continued sensitization through either physical or virtual or whichever means. We need to identify resources to support the provision of those services because quite often students may get prescriptions that are not readily available in the hospital and need to find ways of accessing them.*” (Interview 4, Administrator)

All participants also acknowledged the multiple challenges related to accessing available services. For instance, students reported underuse of existing services whereas the administrators appeared to be struggling with how to help students. In contrast to this, the counsellors described being overwhelmed with no special designated area or space for mental health services. In response to this confusion, students described taking the initiative to start up mental health service delivery groups:

“*I have seen a number of students come up with many campaigns regarding mental health.*” (Interview 9, Student Leader)

##### Peer to peer support for mental health

The process of obtaining mental health support was described as laborious, lengthy, ineffective, and inefficient. Given the challenges with the mental health support system for medical students in the university, all participants described relying on a peer support system to address the mental health needs of students. Participants described appreciating the effect of the peer support system in assisting the university offered mental health services. For this reason, peer support was promoted and student leaders were trained to offer the service among their colleagues by the university counsellors. This one-week training transformed students into a pseudo role of a fully trained counsellor. However, in case these student leaders faced challenges with the cases that they had, they were supposed to refer to the counsellors and administrators. As mentioned previously, many students had decided to form mental health groups to help others with mental challenges to which the university was grateful for the assistance offered. However, the peer support system was associated with challenges such as compromised confidentiality, which was described as a significant detractor from accessing mental health services. The peer support system also comprised a group of students who did not have professional training in the management of mental health challenges:

“*I know there is peer to peer support. I have quite often listened to students trying to support their fellow students who have got some mental health challenges. I have received students coming to my office telling me of how they are trying to help their colleagues to access psychiatrists and pull through some of the challenges they are facing.*” (Interview 4, Administrator)

“*Mental health is not something you can easily just refer, because you are dealing with someone’s confidential issues, you’re not sure if they want to go somewhere else. So, maybe I can understand why people (student peers / leaders) try to help you by themselves, but I feel we should really work with other especially more qualified people to see that we help the students that come to us.*” (Interview 5, Student Leader)

##### Waiting for a breakdown

Without explicitly saying so, the descriptions of service access referred to a waiting period where the institution waited for students to have an overt “breakdown” at which point it was obvious that there was a psychiatric problem which required a medical intervention. For example, students reported a series of process steps which included speaking to a psychiatrist about a fellow student as one way of accessing help, even without consent or permission. At times, this would allow “forced” hospitalisation for the student. Students described witnessing very sick colleagues and the way in which they were publicly “apprehended” because of their mental illness, which caused trauma. It also resulted in serious resentment towards the psychiatric ward and its services, thereby increasing stigma associated with mental illness:

“*There is a student who came here [from a referral from peers]. I told her to go to the psychiatry ward and I think she almost hated me for it. She actually didn’t go and came back here when she was kind of worse. When I asked her if she went, she said, ‘No, I could not.’ I actually forced her to go.*” (Interview 3, Administrator)

“*Many times, with the support of fellow students and the university security teams, we have to actually force them to go or work with the psychiatry unit to actually handle some students and force them into the psychiatry unit for care … so, that kind of stigma that initially not many people will voluntarily walk in the psychiatry unit for care because of the thinking mental health is associated with madness.*” (Interview 2, Administrator)

#### Theme 5: Barriers to mental health services

Despite counselling being a key element in mental health services, students perceived receiving any form of care as being labelled mentally handicapped. This was an element attributed to reduced awareness about the services in the region rather than stigma. This was clearly illustrated by the following quotation:

“*In Uganda, counselling is a very young profession and so for that reason when you tell somebody that you need to see a counsellor somebody thinks [they are] mentally sick. And therefore, telling the person you need a counsellor is kind of saying … [that] you are not stable in your head.*” (Interview 6, University Counsellor)

##### Stigma

All participants emphasized stigma’s disabling effect and reported several ways they had tried to combat this. For example, the students described organizing mental health awareness functions and public lectures, in addition to forming mental health support groups. The administrators described attempting to provide special care for students experiencing mental challenges whereas the counsellors described locating in strategic places at the university to help students access services in a way that would not attract a lot of attention:

“*In interacting with the student, you realise this student may be dealing with this loss [of a patient] and probably they are slipping into depression. The moment you suggest that you need to see the university counsellor and you need to be supported and be able to cope with the loss before you even try to concentrate with the exam alone, the student feels you are telling them they are mentally unwell. They feel a certain level of stigma.*” (Interview 6, University Counsellor)

##### Cultural concerns and religious views

Several participants described cultural practises in Uganda as it relates to speaking about personal matters. Specifically, counsellors were seen as being relative strangers and were not perceived as offering therapeutic anonymity. Participants noted that in speaking to a counsellor, privacy was potentially challenged and did not feel that confidentiality would be respected. Participants elaborated on the fear most students had towards the use of mental health services offered by the university, particularly with the counsellors. Cultural views on mental health were also offered as it related to religious views where sharing of thoughts and feelings would be seen as a sign of weakness. Additionally, a cultural belief of mental illness having a spiritual aetiology was common. This is illustrated with the following quotations:

“*I think that most times we have a culture where talking to a stranger is not something that is commonly done in the African societies, especially if you come from different cultural backgrounds.*” (Interview 6, University Counsellor)

“*We have the different culture and so the culture itself is also another big hinderance of counselling services and then the religious belief. For example, if the counsellor is a Muslim, sometimes you [student] say, ‘This is a Muslim, I am not going there.’ You forget that this is a professional service that somebody is going to give.*” (Interview 7, University Counsellor)

##### Privacy and confidentiality

The students believed that information about their mental issues was tracked on their academic documents, which was confirmed by the administrators. The administrator kept records of students who broke down and also informed their families about the incident. Students did not feel comfortable with this, and they felt it was a breach of confidentiality making many shy away from using the offered services:

“*When a student breaks down, we actually go to their file, check for the next of kin, call that person, and if possible, encourage them to come to let them know what is happening and engage them as well.*” (Interview 3, Administrator)

“*Most people would prefer that their situations or their issues remain private, so that also really discourages getting help.*” (Interview 5, Student Leader)

## Discussion

Despite many studies conducted about students’ perspective about mental illness [15, 30, 31], no study has previously been conducted to assess perspectives related to mental health services offered to students. This qualitative study described the perspectives and experiences of student leaders, university administrators, and university counsellors with the mental health services offered at the MUST in Uganda for medical students. These perspectives were described in five broad themes: (1) Burden of medical school: A curriculum of trauma, (2) Negative coping mechanisms and the problem of blame, (3) The promise of services: Mixed messages, (4) A broken mental health system for students, and (5) Barriers to mental health services. In answering the research question, the data obtained from the three groups involved in this study revealed some contradictions and set the stage for this discussion. In general, administrators and university counsellors reported that mental health services were readily available to medical students whereas the student perspective was in direct contradiction to this, underscoring a tension that was present throughout the interviews between the groups. One of the conflicts that emerged from the discrepant perspectives was the problem of blame where students described a university mental health system that failed to address their psychological distress and mental health needs whereas the university administrators and counsellors suggested that by not making use of the services and for turning to other coping mechanisms such as drug and alcohol use, students were to blame. This problem further reinforces the lack of clarity and uncertainty about the effectiveness of the offered / available services.

In this study, the voice of the student leaders articulated an experience of suffering and stated a need for both an understanding of the origins of their distress, as well as the need for mental health services. However, the different perspectives reflected in the interviews demonstrated that there were differing interpretations of the problems or lack of problems at hand. The counsellors may want to show how much work they are doing or demonstrate knowledge about the idea of counselling to ensure job security. University administrators may want to highlight the effectiveness of their planning and administrative capability, thereby mentioning only the positives about the system. Despite the different perspectives, most participants observed a broken system in the university to support the mental well-being of medical students, whereas a small group of administrators believed the system to be functional and effective.

Ugandan students come to medical school with a lot of pressure on them given that medicine is a course for the brightest youth in the country. They are pressured to pass and meet the expectations of themselves, parents, peers, and government [32]. Failure is never an option. This causes significant distress and pressure to perform as expected even before they start medical school. With no proper mental health system to handle such distressing events, coupled with other psychological distresses encountered by students in medical school, such as witnessing death for the first time [1, 2], puts many students at risk of psychological or mental struggles. These struggles lead to many leaving medical practice after medical school to join other professions, such as business [33, 34]. However, not entering practice after completing a medicine is considered a failure since medical school is an opportunity to build a better life and future, especially in the struggling economy of Uganda [35]. With all this psychological suffering and pressure on young students, they are fragile and need to be handled with absolute care for the betterment of the future health system.

More recent medical education literature suggests that historical attempts to develop medical school curricula must focus on the mental health of students [36]. The nature of medical school training [15] requires students to be in situations of human suffering that affect them, as well. These situations have been widely described as being associated with the development of psychological distress as well as potential anxiety, depression, and burnout being a part of the learning experience [11, 12, 14, 22]. However, in the observations made by the counsellors and the administrators, there is a conflation of psychological distress and psychiatric disorders, which is understood implicitly by the students. Given the profound stigma that exists in Uganda around mental health disorders, a hidden curriculum of suppressing thoughts, feelings, and reactions to potentially psychologically distressing or traumatic situations emerged for the students. Otherwise said, the hidden curriculum tells students that their feelings are best left unspoken and dealt with by employing a “stiff upper lip.” In turn, there is an unspoken expectation that students deal with the routine psychological stressors experienced in medical school precisely because they are common and expected as part of the experience. It is interesting to note that some of the participants representing the administrator perspective were physicians and would have lived experience with respect to a medical school curriculum marked by hardship. These views about the “typical stressors” such as exposure to dying patients and situations where interventions are futile because of lack of resources endemic to public hospitals in Uganda have likely contributed to a lack of curriculum development to support students in these situations [37]. Experiencing these kinds of events can therefore lead to significant vulnerabilities for students with respect to their mental health, where the implicit message is “deal with sickness but don’t get sick.” However, mental illness is increasingly being recognized within the medical student population [11, 12, 14, 22]. In this study, any Ugandan medical student willing to acknowledge that they may have a psychiatric condition requiring help becomes a potential for serious stigmatisation, since mental health services are considered to be for the seriously mentally ill in Uganda [38].

In addition, students who turn to the use of alcohol and other illicit drugs as coping mechanisms to address their psychological distress are pathologized and criminalised. The issue of criminalization of drug use is a complex one in Uganda given that illicit drug use (except alcohol) is considered illegal in the country [39]. The medical culture intersecting with the socio-political context creates the “perfect storm” where the only kind of student coping that is not punished is silence, even if the silence worsens fear and distress [40]. Suffering often requires a voice in order for it to heal [40], and in order for this to happen in a natural way, a model of peer support seems to, at least on the surface, make a great deal of sense to implement as one strategy to support student mental health. Peer support models for mental health have been used in Ugandan medical schools since 2002 [22]. The phenomenon of peer support models emerged in this study as the most relied upon format to address student mental health. Students were introduced to mental health services offered by their peers, a method used due to the understaffing of the whole system, and the few mental health workers available would wait for patients to have broken down before being seen, often leading to severe consequences, such as forceful removal from the public and management in psychiatric facilities. The preferential reliance on this model appears to be in response to inadequate mental health services. This sets up a scenario that requires student leaders who are already suffering to not only observe their peers undergoing traumatic psychological distress but also be responsible for addressing peer distress when they are themselves distressed and not qualified, thus leading to the aforementioned consequences. This finding resonates with other studies,  since exposure to people with psychological distress or trauma is considered "contagious" to less experienced people / therapists [41, 42]. The poorly structured system to handle student psychological distress leaves many of the peer support team exposed to peers with psychiatric conditions, thereby worsening the mental health burden within the student groups and leading to more students diagnosed with psychiatric disorders.

Culturally, Ugandans are typically expected to be mentally strong and it is not easy for an individual to open up about their psychological suffering [13]. The medical culture and its labelling of mental health problems and services supports a stigma that contributes to the service underuse, as reported by participants in our study. The poor use of services is further affected by the university having a mental health service system designed to handle students who have mentally broken down. For example, the university has the university security ready to take and force students to the hospital psychiatry department / hospital in case of a breakdown. This inhumane handling of ill students labels mental unwellness a handicap and increases the prejudice towards mental illness in the country. In turn, the loss of liberty due to being labelled mentally unwell leads to being treated like criminals or unwanted individuals in the university environment, which has been reported by other Ugandan researchers to lead to greater levels of stigma [38]. Mental patients in Uganda, have been handled in various inhumane ways such as being put in seclusion rooms during admissions, tied like animals, and beaten by the community, mental health staff, police, and families [38, 43, 44]; this makes people hide when they have psychological distress or any mental symptoms. Universities, as being the highest institutes of education, should have a different approach to handling students with mental challenges, such as designing curricula that promote mental wellness (i.e., multiple breaks, mentorship, routine meetings with university counsellors), create safe spaces for mental health where people can practice mental wellness practises, and create cultures / systems where students and practitioners can talk openly about hardships, failures, etc. [3, 4, 45, 46]. Such methods can reduce psychological distress and can provide an opportunity for the early detection of symptoms.

Since colonial times, there have been grave differences and poor relations among people belonging to different religions / culture in Uganda [47], and there is disagreement about many perspectives in life, including mental health. Various religious denominations have emerged, with contradicting approaches to mental health. Some religions hold the belief that mental illness is work of the devil, and can be healed only through prayer [48]. This has led to many students being taken to churches for prayer, which can delay proper medical or psychological treatment. Researchers have developed interventions to overcome this barrier, but sustainability and scaling up to involve many cultures and religions is still a challenge [49–51]. For better student mental well-being, approaches to challenge religious and cultural barriers to proper mental health care should be incorporated into students' mental health services.

## Strength and limitations of the study

The main strength of this study was that it employed a qualitative approach to explore the perspectives of different mental health stakeholders in the university and the related narratives of how these participants understood the underlying mental health problems for students. This contrasts with many quantitative studies, which may fail to capture the detailed representative data considering the different perspectives of the stakeholders. This made it easier to understand the situation at hand and the complexity of the current situation at the university. Secondly, data were captured from the second oldest and largest medical university in Uganda, which may be representative of the situation elsewhere since universities in Uganda have similar curricula. However, this approach posed some limitations. For example, the information provided by the different groups could have been affected by their position in the university, which may have resulted in a fear of being fully open about all aspects of their experiences.

The study had low numbers of participants representing the different targeted groups, partly due to the small numbers of people concerned with mental health care in the university. Although generalizability and reliability are not explicit goals of qualitative research studies, we recommend that future studies involve a larger number of stakeholders from other universities to help ensure the credibility of the findings.  

## Conclusions

Based on the findings of this study, perspectives about mental health services for students in Uganda differ depending on the groups interviewed. However, distinguishing between psychological distress that is anticipated because of the subject matter in learning medicine and identifying those students that are suffering from untreated psychiatric disorders is an important conceptual task for universities. This can be done through offering psychoeducation for administrators, giving arm’s length support for students, and a proactive, not a reactive, approach to mental health. Additionally, there may be a need to re-think the undergraduate medical curriculum so that it intentionally seeks to name and describe the medical culture that impacts learning and to pedagogically prepare students for trauma and its personal and professional impacts.

## Supplementary Information

**Additional file 1.** 

**Additional file 2.** 

## Data Availability

The datasets and transcripts used during the current study are included in this published article as Supplementary File 2.
