# Supplementary material for: Perspectives on mental health services for medical students at a Ugandan medical school
Source: BMC Med Educ. 2022 Oct 25;22:734. doi: 10.1186/s12909-022-03815-8 (PMC9592876; doi:10.1186/s12909-022-03815-8)
Supplement: Supplementary file 1 — Additional file 1. [file 12909_2022_3815_MOESM1_ESM.docx]

**Supplementary Material 1**

| **Introduction Key components.**   - Thank you - Your name - Purpose and procedure. - Confidentiality - Duration - Concerns. | Thank you very much for accepting to participate in this study.  My name is ………………………. and I would like to talk to you about your mental health and the mental health services offered by the university. We would like to know your experiences and perspectives towards mental health services offered at the university.  The focus group discussion will take about 30 minutes. I will record the session so that nothing is missed out. I will also be writing down some important information.  All of your responses will be kept confidential and only shared among the research team. In our final report no information will be traced back to you as the respondent.  Remember, you do not have to talk about anything you do not want and you may end the interview at any time.  Are there any concerns or questions you have? |
| --- | --- |
| **Background information.**  **Collected and kept before the start of recording.** | Tell me about your self |
| **Exploring the mental health services offered at the university** | 1. Tell me about the mental health services offered at the university?   Probe about   - How students cope with mental illness? - the type of mental health services offered? - If the services are customised to each student’s needs? - Your perspective on the effectiveness of the offered services |
| **What are the facilitators barriers towards access of mental health services?** | What mental health services are you involved in? Do medical students utilize these mental health services offered? Tell me about the barriers towards full utilization of the services?  Probe about:   - If students know about these services? - If students utilise these services? - How often students utilise these services? - Efforts made by university to let students know about these services? - the different barrier towards access to mental health services by medical students? - what has been done to solve them and what they can do to improve the status quo? |
| **Utilisation of mental health services?** | A). are mental health services relevant to medical students? Tell me about their relevancy”  Probe about:   - the different experiences of people in regards to mental health service? - If the services are evaluated on their relevance and importance? - what they would do to improve the services? - How they would improve the services? |
| **Recommendations.** | What things would you recommend to ensure utilization of mental health services in the university by medical students? |
