# Supplementary material for: Perspectives on mental health services for medical students at a Ugandan medical school
Source: BMC Med Educ. 2022 Oct 25;22:734. doi: 10.1186/s12909-022-03815-8 (PMC9592876; doi:10.1186/s12909-022-03815-8)
Supplement: Supplementary file 2 — Additional file 2. [file 12909_2022_3815_MOESM2_ESM.zip › interview 10.docx]

Knowledge, attitudes and perspectives of medical students of Mbarara university of science and technology on the utilization of university offered mental health services

**Interviewer**: yes, my name is **Interviewer**, I am a fifth-year medical student at Mbarara university of science and technology and I would love to first of all thank you very much for accepting to participate in this study. I would like to talk to you about mental health and mental health services offered at the university. We would love to know. Briefly we would love to know about you and your position in the university and then we will also as time goes on, we would love to know your experience and perspective towards mental health services offered at the university. Approximately the interview will take 30 to 35 minutes depending on the rate at which you respond to the questions and the session like I told you is being recorded and I would also do some writing of the important information such that we do not miss anything. All your responses are going to be confidential and they will not be shared with anyone except the research team and none of the responses still will be traced back to you. For example, your name, your class, your year. Those will not be included. So, as you are in this interview, you are free to talk about anything you know and also free not to talk about anything that you do not want. You are also free to end the interview at any time you want. So, are there any questions or concerns before we set off?

**Respondent**: not really, I am ok we can go on.

**Interviewer**: alright I would love my colleague to introduce himself such that we can get started.

**Interviewer**: yes, thank you very much Dr **Respondent**. I am interviewer 7 and we are really glad to have you here. Thank you for your time. We know your time is very valuable and really, we appreciate you so much. Thank you very much.

**Interviewer**: alright, thank so much **Interviewer**. So, **Respondent** we would like to know more about you, so tell us something about yourself and what position you hold in the university.

**Respondent**: Yes, I am a medical student in my final year at Mbarara university of science and technology doing bachelor of medicine and surgery, that is medicine. I am a resident while at campus and I happen to be a minister in the current government of H.E MAI as the minister in charge campus affairs. All matters concerning the university when it comes to campus affairs that is things to do with students and their welfare, I am responsible and to the whole executive. I am glad to be on this platform **Interviewer** and **Interviewer**, I am so glad to be here. Thank you for this opportunity.

**Interviewer**: alright thank you so much **Respondent** for your humble introduction. So direct to the point. I would love to know what are some of the mental health services that you know which are offered at the university.

**Respondent**: so, some of the mental health services offered at the university are, of course when you say university, it is sort of narrow, I would probably say university and hospital. Because most times when we have students breaking down with mental related issues, we tend to involve the university as a whole and the hospital; but still at the university level, we have a counsellor GN as a university counselor. At least personally I have been there for issues concerning personal but still they were basically academic related. Just to go and interact. Probably I felt like I was burning out. The course has been so long and it was becoming intense. I remember my moral for the course was getting off because the course is too much, we have over stayed in school and things are not coming to an end. I mean I needed someone to talk to, I needed someone to encourage me. I needed someone to just motivate me and she took me through and probably I got encouraged and motivated and that helped me to get back. And the other thing is of course the groups within university. The groups or bodies. The likes of the religious groups, then the associations formed by the students in terms of where they studied from or in terms of where they come from in terms of districts or regions in Uganda. Every time they meet of course they tend to encourage one another. They also come and try to relate and probably the superiors, what I mean is those that have come earlier than the others, they tend to probably give help to the colleagues, talk to them how to go through campus, how to manage finances, manage relationships because all those things contribute to mental break down. This act as places of encouragement of course to eliminate things like depression, things like anxiety, things like OCD or mental related. May be the other thing probably some of the course units we tend to offer at campus, the likes of psychology, probably you also get an opportunity to be for someone to really learn and they get to know their personality. So, when things happen, they are not a surprise, at least someone knows their personality, someone knows about their weaknesses and their strength. You know when things are failing out there, I don’t need to stay in my room to burn out, I think I need to look out for someone like a friend or people to talk to. Ibe encouraged, I be motivated by people. When you cross into the hospital lecturers, especially the psychiatry doctors, those guys have always come up to say in case you identify a friend who needs really some mental attention, always come to them. Or if someone is feeling like a burn out, look out for at least a medical student or someone who is allocated at lower campus and they can probably direct you to where you can find better help. I have heard a friend she has maniac and bipolar, bipolar actually is that combination of two psychiatric conditions. She happens to confide in me. Every time she feels a burn out or things are not adding up, she finds hallucinations and so she could come and talk to me and of course I could find a way of translating to the other side of the hospital and I think her psychiatrist was one of the psychiatrists. Then the other thing from the hospital is attending to the students, giving them drugs for those who have mental related things and stuff like that. I don’t know if I have left out anything.

**Interviewer**: thank you so much. So, tell me a little bit you said you have been at the ministry of campus affairs, probably some of the mental health related conditions or services that you have probably been able to interact with have come either when you are the minister of campus affairs or when you were a student before

**Respondent**: It has been a mash up, it happened before I was a minister, and it is also happening when I am a minister. But previously I have been a student, I could utilize the position of being a medical student to

**Interviewer**: sorry we had lost you a little bit. I wanted to inquire based on the time that you have been able to offer these services, what has?

**Respondent**: sorry because of the network. I said before I became a minister campus affair, I utilized the opportunity, sorry I had lost you, pardon me

**Interviewer**: so, during that time when you were offering these services, what has been your experience regarding student’s mental health and the mental health services offered.

**Respondent**: so, one, one of my greatest experience I came to realize there are so many students out there. Probably when I say out there, I am assuming people are so out there, probably in their hostels, in their homes and in different places around where they stay while at campus and many of them are breaking down out there. One, many are naïve, two, many are shy, three, many are antisocial which happens to be one of the mental issues, then the fourth part would be to identify the rightful people that could be of help. Why am I saying this? I remember way back in my second or third year while I was staying in katete happens to have come across, actually I remember it happened during the first lock down, was it second, not really, I can’t remember the exact time. But this student was offering as course related with computer either computer science r computer software, something like that.so we get a report that this student, previously before she even, of course I will not disclose, before even she thought of hanging in the room, she had things that were ongoing probably home related and had or is being heat by pressures both from home, and studies, probably someone has a relationship. This person was carrying luggage of issues and this pressure became so high and this student resorted to hang herself. So, it simply meant that probably if this student had the rightful friends to talk to or the rightful people to approach, I am very sure this life could have been saved. Because we understand very many people go through a lot of challenges while at campus. Not everyone finds tuition on time before exams. Not everyone finds pocket money on time before hunger strikes in. I mean a lot of things; you find that you get to campus but we are from different back grounds. But I am very sure I know at campus there are these small associations that tend to come up to help students probably on things to do with tuition, food and stuff like that. So, I feel like many out there they really don’t know the rightful people to seek for help from. So that was one of my greatest experience. But what I once also Identified on the services provided surely these lecturers or the psychiatrist, they are very interested in the mental health of the students because I remember one of the lecturers that was lecturing me. He said incase you come across anyone, or incase you identify anyone, get them to us or connect us to them so that we could be of help, so they are really good people who are actually willing and they are very interested in the mental health of students while at campus. Because they also know, they have been doctors of medicine. Of course, the other courses are stressing but they have been doctors so they know how stressing medicine is and so they would really run very fast and try to help out. May be the other experience, I remember one of the ministers, the current prime minister has a small group where she meets up with, she formed a small group and those are ladies within the ladies’ flat. That they gather together from different years now these are only medicine students, you can call it a focus group discussion. I think I happened to have been in one of those groups that was in one of the discussions. During the previous lock down, I had a mental burnout because of the course and the pressures from home. But this student happened to have landed into the hands of the doctor AS and Dr AS would encourage this lady and currently this lady is recovering. yeah, surely the services are good

**Interviewer**: alright thank you so much for that submission. Now I would like you to tell me about the types or categories or examples of the mental health services offered at the university.

**Respondent**: yes, types or examples of mental health services provided at campus. Ok, so, one of course when you talk of mental health, of course you can’t leave out the other professions that provide these kinds of services and among the many of course you think of the psychologist, counsellors, clinical social workers, occupational therapist, then, those are the people. I think they provide services like of course now the psychiatrist has to, probably if they get down someone, they can probably utilize interventions such as cognitive behavioral therapy basing on how someone presents. I think the presentation of someone is very key to dictate how the intervention is going to appear like. Imagine if someone just has anxiety

**Interviewer**: hello are we still there. Sorry my zoom went off abruptly, are we still there? Are we still there? Can you hear me
