# Supplementary material for: Perspectives on mental health services for medical students at a Ugandan medical school
Source: BMC Med Educ. 2022 Oct 25;22:734. doi: 10.1186/s12909-022-03815-8 (PMC9592876; doi:10.1186/s12909-022-03815-8)
Supplement: Supplementary file 2 — Additional file 2. [file 12909_2022_3815_MOESM2_ESM.zip › interview 11.docx]

Knowledge, attitudes and perspectives of medical students of Mbarara university of science and technology on the utilization of university offered mental health services

**Interviewer**: You are welcome **Respondent**

**Respondent**: good afternoon

**Interviewer**: good afternoon to you. I am trying to reach this other colleague of mine, he is supposed to have logged in, ok, alright. I hope you are having a good day prof

**Respondent**: yes, yes, yes, of course there is a lot to do and there are supposed to be many meetings, and there will be another one after this one so it is like being busier than when you are at campus.

**Interviewer**: I understand, I understand, we will actually keep this brief and short, yeah. So, for the purposes of formality, my name is **Interviewer**, and I am a 5^th^ year medical student at Mbarara university of science and technology. I am part of a team that is conducting a research study to assess mental health service utilization among university students at Mbarara university, and I welcome you to this key informant interview. Thank you for accepting to participate

**Respondent**: you are welcome

**Interviewer**: yeah, the interview will take about fourth minutes and this session is being recorded such that nothing is missed out. I will also be writing out some important information and hopefully your responses will be kept confidential and only shared among the research team in our final report. No information will be traced back about you as a respondent. Yeah. You also have the freedom not to talk about anything that you don’t want and you may actually stop the interview at any time.

**Respondent**; ok

**Interviewer**: are there any concerns before we can kick off?

**Respondent**: yes, what is the purpose of collecting all this data?

**Interviewer**: the purpose of conducting this data, there is a research study that we are conducting and we believe that

Participant: yes, how will you use the results, that is the relevance

**Interviewer**: the results will help in the formulation or drafting a manuscript, which we will publish in an international peer reviewed journal which we hope that it will help in policy implementation and assessment regarding mental health service utilization at our university and across the other universities

**Respondent**: alright, that is ok I think that is a very appropriate answer, so you hopefully use this to help us to improve access to mental health services by students

**Interviewer**: exactly

**Respondent**: alright, go ahead

Interviewer 5: so, tell me a little bit about yourself, this could be in form of the position you hold at the university or in the hospital and specifically in the department that deals with mental health.

**Respondent**: So, my names are **Respondent** and I am an associate professor of psychiatry at the faculty of medicine, department of psychiatry, Mbarara university of science and technology. Though we do not have formal appointment letters, though I don’t have it, but I am also the psychiatrist for, in the hospital. Because ideally in the hospital it is supposed to be an appointment to people who teach and also vice versa. If was at the hospital, I would be a senior consultant

**Interviewer**: ok, thank you so much, then I would also like to know about the utilization of mental health services by medical students. For how long have you been in the mental health service and regarding the utilization process by medical students, how has it been, how are you, tell me a little about that.

**Respondent**: yeah, so I have been in mental health services from 2000, all about 21 years, but in Mbarara from 2005. That is when I came to Mbarara university and I was the first Ugandan psychiatrist to work at MUST (Mbarara university of sciences and technology) and MRRH (Mbarara regional referral hospital). So, the question you have is how is the utilization of mental health services by medical student. So, there are supposed to be a number of ways in which medical students should utilize mental health services. So, one of them should be in the university clinic with the nurse in the university clinic. Unfortunately, it seems in my own rating it is a service that students really don’t uptake. The other one is of course the department of psychiatry. The students, the medical students who are seen at the department of psychiatry are those that are very sick and require admission and that is a bit unfortunate. It is like until when they are very sick, then they don’t come. however, there are some students who are referred to us, myself and fellow psychiatrists. There are some that are referred on individual basis, for instance a member of stuff could be having a medical student whom he/she knows and they think that they are not mentally well, so they contact one of us and they say hey please help us to review this student. So those may not need admission necessarily or sometimes some students refer their friends and say let’s go and see one of the psychiatrists at the hospital, then they come. There are also times when the groups that are rotating in psychiatry. You find that the medical students in fourth year are undertaking a clerkship in psychiatry. So, after learning about the signs and symptoms of mental illness, so then they bring a friend and say it seems you are not well, can we go and Dr **Respondent**, and other doctors see you and I have also seen a few students who do the clerkship and then they come back and say but you know I am on treatment for mental illness and you know I would like you to help me. Then there are also some very few cases where parents when their children are coming in first year and they have been treated elsewhere, mainly in Kampala and now that the student is being admitted to the medical school in Mbarara, so they look for mental health workers within the university and somehow it ends up in our hands, we the psychiatrist, the person is introduced to us and we start looking after them.

Interviewer1: ok. That is so comprehensive.so how has your experience been over the years regarding these mental health services. Like how have you found it over these years

**Respondent**: so, the general trend is, I really think that the mental health services are not uptake adequately by the students who need it. So, what I mean is there is stigma for coming for treatment despite the fact that we are available to help. In other words, there is what I would call a mental health gap. There are many who I would think need the services, but we just see very few. So, there is that gap and so, but also, I am seeing that us on our part we may not have utilized all the opportunities to make the service accessible to the students. Because for sure our students are literate and they have technology which they can use. So, we have not for instance put out some tools online which a student can use by themselves and come out with a possible diagnosis by them themselves, like a screening tool; something like that, which could be available online, so the uptake is not as good as we want. Yeah, however it is like there are more and more students who are being seen much as the number is low, but the number is more than that of ten years ago. Yeah.

**Interviewer**: alright. Thank you so much about that. So, I would love you to tell me a little bit about the mental health services offered at the university, the types of mental health services offered, and whether these services are customized to student’s needs; like if they are offered, do they address the needs of the students specifically or they are offered broadly. And then also from your experience how have you seen the students cope up with mental illness.

**Respondent**: so, what are the types of mental health services. You can categorize into outpatient and inpatient. That is one categorization.so as I have told you before, most of the patients come in as inpatients that means that they need to be admitted meaning that they may come in when the sickness is very severe. But coming in as an outpatient is not very common unless it is a follow-up like we have already seen them. But somehow, we do not put students to come in like other patients come, like they come in, sit on the bench, and wait for a clinician. We do it privately, for instance if there is a student I am looking after, they make an appointment and I see them in my office, yeah so it may not be that it is customized for the students, however, we give them that treatment like colleagues, we take students like our colleagues, they are students, but then they are health workers in training. So, there is no way for instance I would make a colleague who is a doctor already, come to psychiatry department, then see the nurse, then the nurse looks for the file, then he begins queuing until the nurse says go to see the doctor. It is more of a private so that the psychiatrist and the agree when, and sometimes where. I have sometimes seen students in the office rather than in the clinic. They may prefer it that way and say ident want to come to clinic anymore, and say can you see me elsewhere. I can say that is ok, can you see me in my office in the afternoon at 2:00 p.m. so there is no stigma any more, they are coming in like a colleague and then I have time with them. That is how we have managed to deal the students. We don’t want to subject them to the routines of the psychiatry ward. But if it is for admission, there we have no way because we cannot have a separate for them

**Interviewer**: so, you talked about the categorization of the types of mental health services as outpatient and inpatient, could you a little bit elaborate more shortly about the different services that are offered.

**Respondent**: so, we, the services we do offer are, we offer medications; in terms of forms of treatment, so we do offer medications, so that means we are giving psychiatry drugs, and that is a service. The other one is psychotherapy; psychotherapy means talking therapy. that means you are going to look at the strength, weaknesses and the resources the person has and occupationally and look at how they are studying, then you offer counselling. But we have a clinical psychologist who does that. That is his specific purpose.so he might use personal therapy, or CBT (cognitive behavioral therapy). Then the other one is social. Social means, because some students come, because of social issues, could be relationship problems, some students have miss used their tuition, some students have come from difficult back grounds. With completion students, completion students are more mature than the direct entrants. A good number of them have marital problems especially the females, so she has come to study, she has left the husband and the children somewhere, and then she comes to know that the man has gotten another relationship, then things go wrong. So, in terms of forms, we offer treatment as medication, we offer psychotherapy, which is talking therapy. then we also offer social support.

**Interviewer**: so, my colleagues have something to ask about that section, we will address and then we go to the next questions, I see we are left here with like 20 minutes

**Respondent**: it is ok. **Interviewer** how are you?

**Interviewer**: I am alright **Respondent**. So, I wanted to know like is there a service that the university offers. Because now I know you work for the two institutions, the hospital and the university.

**Respondent**: sorry I missed that, the university has got a counsellor.so we made it known to the dean of dean of students and the university counsellor, that students who think they have any problem, psychological, social, psychiatric or any other problem should go there and there are two people whose position is university counselors. There is pastor GB (George byabagambi), I don’t know whether you know him, there is CK (Cyson katushabe) so those two are recruited as university those two is the purpose. They are supposed to be in the university clinic and do counselling. counselling is normally the initial service that you give to people who are psychologically disgraced should they find that the condition is very severe and needs psychiatric attention, then they would link to us. Unfortunately, students seem not to utilize that service. MK (Maureen Kahima) who is your warden started work as a university counsellor before she was promoted to that position of warden. She also has counselling skills. That is the side of the university. However, I don’t think that we are, we are again, we are not doing enough because when I was the dean of the faculty of medicine, the easiest way to know that a student is struggling psychologically is to look at their performance. I use to call all students who have got supplementary, I would call them to my office and talk to them and find out what the problem is and then there and then you actually find out that they are psychologically struggling. May be the other thing I could give to you is we do write letters for students. This is like a forensic service. A student might have gotten a problem with their studies, but because the university wants to know what the problem is.so we have to provide a letter that says that so and so was mentally unwell and that is the reason why they are not performing well in class and they need treatment and they go back to study.

**Interviewer**: Thank you **Respondent**

**Respondent**: that is on the university side.

**Interviewer**: **Respondent** I would like to know something regarding you and delivery of mental health services. What mental health services are you directly involved in as you, personally. And have students been able to benefit in these services which you are involved in?

**Respondent**: yeah, certainly of course my primary responsibility is to train, to train students in psychiatry and me at both post graduate and undergraduate levels. So, the topics I like much are addiction psychiatry, the reason is substances of abuse are one of the reasons why students fall into trouble in their studies and therefore their future. So, when I teach addiction psychiatry, I make sure that I point out the dangers of drinking or using cannabis or any other drug of abuse by students. And some student shave come there after seeing and they are like I think Dr participant, I think I have a problem of drinking, so how do we, how can you help me, so I pick up from there. Then also the other topics are Gaetric psychiatry, the interface between medicine and psychiatry and then the clerkship in the ward. So, through training students, we give them knowledge in helping others but you also give them knowledge to help themselves, because it is no good helping others when you cannot help yourself. It is of no-good waiting to help patients out there when you cannot actually help people closest to you, like your fellow students. So, there is that training. then the other one is research, both of you know that I am a mentor and I have worked with you to do research in HIV but indirectly mental illness as well. So, there is research which I do but of course through publication and things like that, it benefits the wider community including students. But in terms of care, as I have told you we do look after students, and I have students that I am looking after. There are some students who have, unfortunately they are living with HIV and sometimes they struggle psychologically.so when I come across such a student, I would really help them in terms of taking their medication and in terms of having positive attitude to life despite having a chronic infection which actually has no cure as of now. But hopefully in the future it will be there. So, I do also provide care to students. But also, I do motivational talks once in a while. I remember I gave a finalist talk, that was I think way back about 4 years ago about the opportunities in the career broadly but specifically in mental health. So, we do what I would call mentorship. Like I do mentorship and post graduate students come back to come back to do psychiatry. There was one called NL, she finished her MMed Psychiatry and she is now posted in masaka. So, she is very happy that I talked to her to come and do psychiatry.

**Interviewer**: thank you prof. I would also like to ask. What are some of the efforts that you know that the university has put in place to let students to know about these services. And possibly some of the barriers which you have seen to the access of mental health services by medical students,

**Respondent**: I think the dean of students perhaps is the one who has tried, the office of the dean of students has tried to make these known to the students during orientation. But it is still not so much, it is minimal, the barriers are really the stigma towards mental illness by students and also by stuff. So, nobody wants to be known as somebody who is mentally unwell. That is very unfortunate. Then at one time we had allocated Dr SA to be the direct link between us and the students. But still, it did not happen. It did not work out very well. Although once in a while people would come here and there. But it is also not done very well.so barrier number one is stigma, barrier number two is that, I don’t know whether we should call it a barrier, but we haven’t been proactive to really have something for students. Many universities have a well fare office for students and we don’t have that at Mbarara university. Because if you have a well fare office for students, so then whenever you think you have a problem or something you go to that office. When you get a financial problem, be it a social problem, these things lead to mental illness. But there should be an office to help students who are struggling. And they are helped. So that is the second barrier. So, we don’t have the wellness office for students which should have been ideally in the AR’s office. And then with a department in the dean of medical school’s office, so that students can go there. But the other one is we don’t have a formal mentorship program I the university, and this is a very big barrier. Because if every student has or had a mentor, whom they stat with from year one, this mentor would do academic mentoring, but they would also do life and a balance between life and career, academic career, this person is more senior and has gone through the program, so they can help a student, socially, psychologically, emotionally as well as academically. Then the student has got somebody to run to whenever they have got a problem. However, it MUST in the medical school right now we don’t have a formal mentorship program, which some universities have. By the time you come, you have a mentor. You give your information about your self to the mentor, he comes to know you, where you come from, your family and what you think your challenges and then academically he wants to know what your interests are, what you want to become after medical school. That type of guidance would actually help many students. So, if the student gets into a psychiatric problem, the mentor will be quick to know because he knows the student and they have developed the relationship. That type of gap is a huge barrier

**Interviewer**: thank you so much prof. now I want you to, I believe this will be our second last question I want you to tell me about mental health services and their relevance. Are they really relevant to students, are they really important? What do you think can be done to improve these services personally as you if you had the capacity? How would you improve these services at the university? I think you can use like 3 to 4 minutes then we will go to the final question.

**Respondent**: so, the question is whether mental health is relevant is a relevant question. You all both of you are medical student and you know very well that there is no health without mental health. That is W.H.O. there is no way you can have a productive student when they are not mentally well. It is just can’t happen. Reason is medical school is the most stressing course. Training as a doctor is the most stressing course on earth. If you do studies you are going to find out. You find that medical students are the ones who are most stressed. The curriculum is so tight, you are studying from eight or even seven. You go to the ward. In the ward there are many challenges, you are going to see sick people, you see going to see people who are dying. And then there is lots of work to read. Some courses are tough and so medical school is very stressing. Because of that, many students may not be able to cope with those stresses when you add those to the other stresses elsewhere. So, to make students study and become proper professionals you need to take care of their mental health. Less that, then you are going to graduate people who don’t love the course they are being traumatized, no body is debriefing them after they have seen people who are dying. So mental health is very critical to the health of the students and it is most critical to the health of medical students. And so, if I had resources, I would put into place a mechanism that empowers the medical students themselves to be able to identify colleagues who are mentally unwell. And now this program that helps students quickly whenever they are sick. And in terms of medications, I would really put into place a mechanism that looks for the best medicine for the students, for instance if a student needs an antipsychotic, I don’t see why they should be on chlorpromazine that is going to make them sleep. I would look for atypical antipsychotics which don’t make them to have visible side effects and so a student will not be easily identified by anyone that they are mentally ill. I would also do screening during admission, and orientation you can screen using online tools. Someone can score by themselves. If you score beyond a certain point, we refer you to somebody or someone who can take further from there. So, in that case we would be able to know who is entering medical school and they are not well. But also, we need more on going talks physically and that we can provide, I think I have talked about alcohol, substance abuse, I think this was at FAMSA, something like that, but it can be on a regular basis so that people will come to know about mental health

**Interviewer**: alright, our final question will be, what are some of the things that you would recommend to ensure utilization of mental health services in the university by medial students, especially like in this limited setting, like our country, or a developing country. What are some of those recommendations that you would give out to the medical students or to the university to ensure that they utilize these mental health services

**Respondent**: so, I would really recommend one thing. We need to use the students themselves. We would encourage students to understand what they want. or what they want in place so that they would better use the mental health service. Until when you understand what is prohibiting them from using these services, then improving their utilization will be very difficult. Of course, first of all the services must be available and that is not enough. Think you have seen scenarios where you have a vaccine for COVID 19 and people are running away from it. So, for us to greatly improve access of mental health services, two things; make the services available, but more importantly stick down with the up takers of the services and ask them in which form do they want it, when, at what time, maybe you are going to tell us, maybe you would like to see the psychiatrist at midday, during day I don’t want. Something like that, stick to students and find the best form in which they want the service to be so that they can use it. So, they might say we want it outside the hospital. If you put it in town, then we can come there. Then we look at that. That is what I would do. I would try to find out what do the students want and how can we make these services to be utilized

**Interviewer**: thank you so much professor for your time and we gladly extend our appreciation to you for having accepted to attend this interview. We apologize because of our current status as students we are not able to provide any refreshments at the end of the interview. Thank you
