# Supplementary material for: Perspectives on mental health services for medical students at a Ugandan medical school
Source: BMC Med Educ. 2022 Oct 25;22:734. doi: 10.1186/s12909-022-03815-8 (PMC9592876; doi:10.1186/s12909-022-03815-8)
Supplement: Supplementary file 2 — Additional file 2. [file 12909_2022_3815_MOESM2_ESM.zip › interview 8.docx]

**Interviewer:** So umm…for starters ahmm…I just want to find out a bit yourself and the role that you play towards the mental health of medical students so any role you play as regards utilization of mental health services.

**Respondent:** ...yes…mental health is an area I am really interested in and I have always as a a student I have always been touched by how many of my colleagues have mental health issues and most of them do not recognize that they have mental health issues. A case in point, most of the students that I have encountered that have mental health issues have actually bad coping mechanisms. So, some resort to either drugs others resort to ahh…ahh…unexplained activities…uhh…or indiscriminate sexual activity or certain things like that and it really hurts me that very many people in the university instead of facing their mental health issues resulting from their social and academic lives, they tend to resort to coping mechanisms that are not sustainable and may not stand the test of time. So as a student I have always been touched and I have always looked forward to…so I was saying students have poor coping mechanisms as regards the mental health challenges they face. So, I have always wanted to personally have an initiative that aims to help students not only help [inaudible] …but also be able to access care as regards to mental health. We have always tried to engage the Dean and the Dean’s Office to help us as medical students avail us with, with services because we have a counselor that is almost non-existent and doesn’t help us as students as much as we would like. And also, I do not think the services that are available at the university are centered to benefit the student. Basically, not student centered, they are mainly there for showbiz. So, I agree that the services are not adequate but that doesn’t mean they are zero. So I agree we have where we can start from, we have where we can meet each other, we have where we can…we can support each other, we have where we can leverage the existent channels so that we can benefit and personally I remember, last year during my third year...the toll of pressure was a lot and some people resorted to drugs and all that and I had my own tolls of overweight, mainly academic but also from outside relationships and all those pressures that come from academics from dating, you always feel you are overwhelmed, you always feel like you are eminent for a breakup...for a breakdown but you have no access to help so…or you don’t know who to access for help so the levels of stigma are really high so to some people they are not aware of the services or they can access this help…some people are in denial about needing help. So, it is a whole novel area that I am really help, that this initiative is there out to seek opinions and see how best they can address it. And as a leader, I’ve happened to interact with students that want to commit suicide, not once not twice, there’s even a week we had two girls in second year that wanted to commit suicide. We have students that are on antidepressants and anxiolytics. The scope of mental health at the university is really [inaudible]

**Interviewer:** So, thank you, I just want to find out from you apart from the university counsellor, are you aware of any other type of mental health services offered for medical students?

**Respondent:** Ahh…the students that are passionate…I don’t want to use passionate…that are bold enough can approach the psychiatry department and access help. But those who are not bold enough and are skeptical of the stigma and the high levels that are associated with the stigma cannot approach the department, the psychiatry department to seek help which leaves a really wide gap that there’s no linkage of care and where there’s no linkage of care, there’s no care existent at all. So, it is only for those that know and most times it is only those that have gotten into a sever case and it is a no-brainer to be taken to the psychiatry department

**Interviewer:** So, for those that do not know how do they cope with the mental illness

**Respondent:** Most of those resort to the illicit or the…negative coping mechanisms, some of them resort to talking to their friends and most of the students suffer in silence, the majority of the students suffer in silence. That is the information I have at my disposal. Most of the boys and girls in the upper years that know about it or that have some knowledge about mental health…still may present in a way that is stigmatized, they crack jokes about it so the levels of stigma are still high so very many people just suffer in silence because they don’t know what is out there.

**Interviewer:** Okay. Thank you. Umm… Earlier on, you mentioned that you feel the services are not customized to student’s needs. Why do you feel this way? Why do you think they are not customized and how do you think they can be better customized to fit student’s needs?

**Respondent:** Firstly, that they they should be, there should be a continuous engagement with students on how best they need, they think they should access help. But firstly, they should make the services more approachable, for example the number to the counsellor should be availed to all students and the counselling department should not only consist of one counselor...should have a university counselor [inaudible] students or peer counsellors that try to counsel fellow students. And it is an information age, we can take it up a notch and have technologies put in place that allow for anonymity and someone can readily share with someone and they don’t have to reveal their identity and they can share get a piece of advice. We could have ahh...avenues to talk about most of the challenges and we could have university engagement uhm with the students as often as we can…we can have podcasts; we can have support groups. Support groups are one of the are one of the things that would reduce the stigma and show us that it is okay to suffer from of these challenges for example when someone goes through a challenge, we have had people wanting to kill themselves from going through a breakup or going through a loss of a colleague for example I almost broke down when I lost one of my colleagues early in third year so I think we need to know that there are other people that have gone through these challenges so we can support each other

**Interviewer:** Okay...umm...earlier you also touched on the issue of stigma and ahh...being a barrier to utilization of mental health services if any that are offered. Is there any other reason why you would think umm...students do not fully utilize the mental health services that are available apart from stigma?

**Respondent:** Umm...I think other than stigma, there’s awareness and accessibility as the other factors that I think. Awareness that so do not know that they exist. Two, some they know they exist but they are not student accessible, they could be accessible to each and every one knowing that the psychiatry department is there, but student accessibility is I think more relevant in the sense that some of these programs need to be tailored to the student for example most students want some sense of anonymity and autonomy and the ethical considerations that will take them, they don’t want fellow friends to find out about the issue. A case in point where we have to think about is that the services are not…people are not aware and also the they are not as student accessible with student accessible in quotes.

**Interviewer:** Okay umm…are there any, do you know of any efforts that have been made by the university to let students know of these services?

**Respondent:** Yeah…umm…the Dean, the Dean’s Office normally informs students every orientation of first year. That’s the only thing I know. They inform them that there’s a counsellor, they inform them that the Dean’s Office is always open. Other than that, it is not a continuous process of engagement.

**Interviewer:** Okay so it’s only when one getting oriented so if they miss the orientation, there’s nothing more that is done. So, do you know of any interventions or that have been put in place to solve this status quo?

**Respondent:** No, I don’t know of any.

**Interviewer:** Or to cover up some of these barriers?

**Respondent:** No, I don’t know of any intervention that has been put up by the university. They accept that the problem exists…umm…but they are not working as fast to have something in place.

**Interviewer:** So in your opinion, what can be done to improve the state of affairs as regards mental health services.

**Respondent:** Umm…Firstly we need to tackle all the three avenues. One is awareness. Students need to know that the services exist or that mental illnesses exists and they need to know that they are in control of their own mental health. For example, we should make maybe accessible the tools of assessment and also we should have continuous campaigns to...curb on the stigma in the long term to know that these conditions exist and it is okay to feel down, it is okay to be depressed and it is okay to go through all this and also we should work together…the university should work together with students to functionalize access to care so that we don’t have late detection of cases and those who have cases can access the counsellor in confidence and sessions should be planned, should have a platform where they should be able to access the counsellor for example on that platform they should be able to schedule sessions with the counsellor through anonymity and...they could be able to meet the counsellor so we should have a platform where can access care through the counsellor and the counsellor should have a team that can link to care because I think that makes it easier through the counsellors office and we should have continuous awareness campaigns that will curb the different levels of stigma but also we should focus on uhm pointing the journey of uhh students that are really…ahh…demanding of them especially the transitioning years especially first year and third year and have a focus on them because we need people are changing from one big area of life to another. Basically, that is it.

**Interviewer:** Umm…we are about to conclude so I want to know do these mental health services for example you mentioned that the university counsellor may not be working as he is supposed to So are you aware of any way in which these mental health services are evaluated on their importance?

**Respondent:** Uhmm…No, there’s no evaluation that I know of. But I think they also realize that there’s a gap. But I think they are not in a rash or they don’t think it is an urgent matter that needs to be handled now because I have tried to interact with them. Some of them tell me your students [inaudible] some of my students use drugs that is why they are having break downs, your students don’t know...they are not, they are just engaging in sexual behavior, your students don’t want to study and they don’t realize that some of these issues are now really eminent.

**Interviewer:** So, you would say they don’t think that the services are very relevant.

**Respondent:** Yeah, for them they think uhhh…it works, they think their system works. That’s the thing.

**Interviewer:** So finally, is there anything else that you would like to recommend to ensure utilization of these services by medical students or by university students generally?

**Respondent:** Ahhh…firstly I think we, I would recommend that they work together with students and also students work together with fellow students to present a united front. Whenever, the administration sees students divided, there’s chance for them not take whatever issue presented seriously. The university already knows there are very many students interested in mental health but how do we present a united front to the university, how do we tackle this problem from the root cause so that we could be able to address even the need of the last student. So my idea is let’s work…let the students work together, let them bring every party on board, let them work together and they can reach out to the university…the university will always support an organized initiative especially if the matter is relevant and they have no choice because they also realize the increased the numbers of student breakdowns are.

**Interviewer:** Okay Okay. Thank you for your time. Unless you have a question or a concern or any last words, I think that is it.

**Respondent:** I appreciate the initiative you are doing. I appreciate the mental health. Personally, I am really interested in mental health and my my…I beseech you as All For mental health and fellow colleagues is...let us all work together and I think most of the people that can add something, let us work together and I am positive that at the end of the day, something that matters and impacts society will come out of this. I really appreciate and commend the initiative and the best of luck.
