# Supplementary material for: Perspectives on mental health services for medical students at a Ugandan medical school
Source: BMC Med Educ. 2022 Oct 25;22:734. doi: 10.1186/s12909-022-03815-8 (PMC9592876; doi:10.1186/s12909-022-03815-8)
Supplement: Supplementary file 2 — Additional file 2. [file 12909_2022_3815_MOESM2_ESM.zip › interview summary.docx]

| interview | category | Person |
| --- | --- | --- |
| Interview 1 | Health care provider | Raymond, racheal, David |
| Interview 2 | Administrator | Raymond, racheal, David |
| Interview 3 | Administrator | Raymond, racheal, David |
| Interview 4 | Administrator | Raymond, racheal, David |
| Interview 5 | Student leader | Jolly, timothy |
| Interview 6 | Health care provider | Jolly, timothy |
| Interview 7 | Health care provider | Jolly, timothy |
| Interview 8 | Student leader | Jolly, timothy |
| Interview 9 | Student leader | Innocent, pius |
| Interview 10 | Student leader | Innocent, pius |
| Interview 11 | Administrator and healthcare provider | Innocent, pius |
|  |  |  |
|  |  |  |
|  |  |  |
