# Supplementary material for: Perspectives on mental health services for medical students at a Ugandan medical school
Source: BMC Med Educ. 2022 Oct 25;22:734. doi: 10.1186/s12909-022-03815-8 (PMC9592876; doi:10.1186/s12909-022-03815-8)
Supplement: Supplementary file 2 — Additional file 2. [file 12909_2022_3815_MOESM2_ESM.zip › interview 9.docx]

Knowledge, attitudes and perspectives of medical students of Mbarara university of science and technology on the utilization of university offered mental health services

***Interviewer: Yes, thank you very much Innocent, Interviewee you are welcome, I am interviewer 2, a colleague to Interviewer 1, we are medical students in 5th year and we appreciate your participation in this and we hope it won’t take long. Thank you very Much.***

***Interviewer: Alright, interviewee thank you so much for joining us once again. I would love you to tell us about your self and what positions you hold in the university.***

**Respondent:** Thank you Interviewer 1, My name is Interviewee 6, I am a student of medicine and surgery year 4 am a GRC medicine and prime minister of the MUST guild 2021. thank you.

***Interviewer: Alright, thank you madam prime minister. I don’t know how they address you. Is it your honor, honorable, I am not so much in the political arena?***

**Respondent:** Right honorable

***Interviewer: OH, thank you so much, right honorable, yeah so, I would like to know your role towards mental health of medical students and the utilization of mental health services around the university. What do you know about it?***

**Respondent:** Well, in the recent months, probably or a year or so, I have seen a number of students come up with many campaigns regarding mental health. One of which is outstanding is the all for mental health and also significantly noticed a number of mental health breakdowns in the faculty. But also, having studied psychiatry, I actually got to know that you can get help from the psychiatry department yet this is something that has always been there yet the students hardly know there is help readily available for them. Also having privileged to get on the guild council, I also got to know that the university has employed councilors whose names I bet not many know, where they sit, not many know, and whether or not people get to reach them for help is also a concern. So, I would think that the services are there and they would be used but the problem lies with how many people know that they are there. Basically

***Interviewer: Alright. Thank you so much. So, during your time of service as a prime minister or as an individual who has probably participated in any other activities dealing in mental health, how has your experience been, tell us a little bit about your experience concerning student’s mental health services.***

**Respondent:** Okay, just a few days after I was voted in as GRC medicine, a girl who stays next door, I reside in the ladies’ flat, got a breakdown at the beginning of the semester, I happened to reside in the same room with another classmate of mine who is also in fourth. So, these most people in lower classes reach out to us to ask about medicine and to know other things here and there. So, this girl comes to us and keeps telling us she was in year 2 semester 1 and she kept telling us that she was told the course is hard. Semester is too hard for her to handle and of course we didn’t take it seriously. We kept telling her you will pass. After a few days we got to know she was admitted to psych ward due to anxiety and she became bad. She broke down, she went home, she couldn’t progress perfectly with exams., they had to give them to her on different days that other student because she was anxious. so that prompted me as a person, I do well because I am the same person who is GRC medicine, yeah, it raised my antennae and I started to think, okay, people can break down as we are here yeah, even though as a leader but also as a person, so myself and my roommate started having a care group of sorts with medical students who reside in hall. So, I know not many people know, because it was intended for its beginning it kept to us not open to many because I believe in being able to have people open up in a small environment in a safe area for them to freely share about themselves. So, we held a number of meetings and the very last one was held on Monday this week and that was meant to have us, our sisters’ keepers. It was typically for female students who reside in ladies flat though we had a few females around and a few females who do not reside in hall. That was my personal initiative for the people I reside with. But on top of that I happen to also have connections with the people lie Kihumuro David, who have been running All for mental health and yeah, I’ve been the guild. We have engaged them, a few of them, several times to have a sort of share, encouraging the work they do, but also work with them to see that as many people as we can, get interested in this and seek help where they can. Yeah, that’s what I can say

***Interviewer: Alright: Thank you Thank you so much Thank you so much, tell me about the mental health services offered at the university in case you know any? The types of mental health services, that you know and whether these students are customized to each student for example if there is a service which is offered, say for example, electroconvulsive therapy, and it is offered like does it meet the student's need, does it give that comfort that you know for a student that they are receiving treatment and if at all you know any other.***

**Respondent:** When you say types, you seem to want specific words, so I will request that you first highlight me the types you want to hear.

***Interviewer: Like What kind of mental health services do you know that are offered, for example counseling, is there any pharmacological therapy that is offered, is there any kind of admission, it could be inpatient or outpatient, yeah. Or like for example if it is inpatient, do they do electroconvulsive therapy, you know, yeah.***

**Respondent:** Okay, so like I said, in my introduction, the university has a university counsellor who is supposed to counsel both the staff and the students in case they have any areas of breakdown. Secondly, even though the memorandum of understanding between the Mbarara Regional Referral Hospital and the Mbarara University is not clear, well at least according to me, but, very many times, we have heard students seek help in the hospital and have been attended to, because many officials or many doctors who are employed in the hospital are also lecturers in the university. So, I want to say the department of psychiatry of Mbarara regional referral hospital is available and offers both inpatient and outpatient care with very very caring doctors. I’ve actually interacted with one or two, they appreciate the services. But the bigger question is are they student, are they student customed. Well, ECT, I just studied it in class, I don’t know whether they do it in MUST. but what I know pharmacology, counseling, pharmacotherapy, all happen at Mbarara regional referral hospital. It is student accustomed but the problem is students do not know that it is there. And that is the gap that we have. That these services are there, people can find them but the problem is we do not know they are there, For the university counselor, I can tell you the truth I don’t know whether anyone has reached out. like students, but they are there. I don’t know if they were student accustomed probably students would know them better.

***Interviewer: so, in other words there is still that gap which is there between student’s awareness of the services offered regarding mental health.***

**Respondent:** Very much

***Interviewer: Alright, so tell me, what are some of the mental health services that you as individuals are involved in for example you talked about the experience where one of your friends was your neighbor got a mental breakdown. So, as you as an individual what are some of the services that you were able to offer to that person regarding restoring their mental health status?***

I'll give one, good therapy, we, I mean that event led to us starting to realize the essence of sharing which happens in a group. The reason as to why she was breaking down, is because she was a medicine student and medicine was weighing her down. So, we decided as medical students. let’s sit and share experiences and encourage one another. So, I can say that mainly group therapy and maybe one on one counseling sessions that are more individual than official.

***Interviewer: So, could you be knowing the frequency with which these students utilize the mental health services, yes, I know you’ve spoken about the gap which is existent, a very big, gap, but then, I believe or I know that there are some who still seek these services, these mental health service in case a breakdown, perhaps maybe, their friends alert maybe the people and they be like, hey tis person is breaking down. Could you be knowing the frequency with which some of these students utilize these services?***

**Respondent:** By frequency, do you mean per individual or general?

***Interviewer: Generally, generally***

**Respondent:** Well, I think people, okay, I’ve also noticed that its mainly the medical students who seek the help from the available resources especially the psychiatry department, because they are the ones who know about it, but also, I received a complaint from the business students when they go, it’s hard for them to prove they are students of must because they don’t get to interact with the hospital often, the frequency is reliant on who has sought the help first. The problem is the initiation. For someone to go first. Those that have gone there before will tend to go there anytime they don’t feel okay. Those that have not gone there, I believe it’s really, it’s not an everyday thing that they get those people going there. I hope that is clear.

***Interviewer: okay, so, apart from the awareness gap which is existent, regarding students knowing these services are available, what are some of the other barriers that you know which are there towards the access of mental health services by medical students?***

**Respondent:** Well, I will start with an individual one which is denial, not many people actually want to concede to the fact that they could be having a mental health condition, so they take so long to know their dealing with all other things apart from the real issue. So there in denial and do not concede and they’ll not go seeking help. The other is the stigma, people just, it is not a common thing for people to you know be diagnosed with bipolar, people start looking at you as a mad person. Like how will you be a doctor eventually. so that kind of stigma is one of the reasons why people do not seek help. and yeah, basically, those are the two that can come to mind.

***Interviewer: Okay, so what do you think has been done to you know to solve the, or what they can do, or what do you think should be the remedies towards those challenge that you’ve spoken about?***

I think I noticed what’s being done regarding mental health awareness, so we are hoping that in the near future we are going to see less people going through denial and yeah, also, because of the mental health awareness, I believe it is starting to get normal for us to know that someone can be depressed and its okay. Someone can be going through this and that and its okay. I think awareness goes to the society, because eventually these people come back to us but also the individual seeking help is the good remedy.

***Interviewer: Okay, Alright, I don’t know whether my colleague has questions so far?***

***Interviewer: No No No No let’s continue***

***Interviewer: Ahhh Okay, so, alright, So, Right Hon. interviewee, I would want to know from you, are mental health services really, really, relevant to medical students? and if so, tell me about their relevance, if not, why?***

**Respondent:** Well, I think they are really, really, relevant to medical students because medicine and surgery, the course and the profession are not very normal courses, normal events, we get into medical school hyped, and you know you are going to be one of the most important people in society and all that you know about it, is it’s a prestige that should come after and unfortunately, when we are being guided career wise, we are really never told what exactly it entails. So, when we get to medical school it gets hard. The content, the course content is hard, what it takes is hard, the ward rotations, the interactions with doctors, the meeting of death, the first time you lose a patient, the first time you see breathe leave someone, those things can get depressing, and also the stress itself, I mean you are around sick people all the time. So, there is a high rate of breakdowns and because there is a high rate of breakdown, then I really think the services are more relevant than we actually take them. I've also heard interactions with people that are above us, those that have finished medical school, those that are doing their postgraduates, and, people are breaking down, yet they look like they have everything together, they come and percuss you here and there, they drive nice cars, but when you get to know them personally, you realize they are breaking down. So, I think that on top of these services being in medical schools, they need to be in all hospitals need to have a provision on which doctors can share experience. Again, not just going to a psychiatrist, but I think some of these things can actually be resolved without them necessarily getting into the extremes for example, just sitting down to share an experience, and say you know the first time I lost a patient, I almost dropped out of school, or I am a doctor and I was dealing with this case and am feeling depressed about it and you laugh about it and take a cup of tea. I think those are things that we neglect but they tend to be more important that we would like to consider. Thank you.

***Interviewer: Alright, so we'll go to our final question of the interview. So far so good. Thank you so much Brilliantly responding to the questions that have been shared. So, what are some of the things that you would recommend to ensure the utilization of mental health services in the university by medical students? Like as you in the position which you are in as right honorable or even as a student, yeah, of medicine. what are some of those recommendations that you probably give out to someone probably those who are in places of authority or those who have capacity to do so. What are some of those recommendations that you would give?***

**Respondent:** Okay, So, I will say knowledge, knowledge, Knowledge, when someone doesn’t know, they’ll probably struggle not because that help is not there but because they don’t know that help is there, so the recommendation is that information is spread. I mean we have social media platforms, we have notice boards and engagements. There are so many university engagements that happen, that people get accustomed. The way we know that there is actually. there is a lot of information gap in the university regarding so many things, but I think that information gap comes back to us as students because when we get into these offices, we get to know things that are not probably common to the usual eye, and I think it’s one of the things that we need to do. But also, not just the guild leadership, but the medical students’ association leadership, the student must know these things, the clinical students who get more accustomed to the hospital and the services available back to their preclinical fellows, so I think, a spread of information but also as that information is spread, it is not biased. The awareness, the mental health awareness, that it’s okay for you to feel this way and when you do that there is someone available and they are going to help you. recently the university was doing sexual harassment awareness kind of thing and even though many students have actually come up with mental health, but I think even the university administration should actually come to realize that this is more than we think, and do more about it. For example, we have a counselor, the first time I got to hear of it, I was even shocked, of course it’s not that I didn’t expect that there is one but the fact that am 4 years in the university, I don’t even know a friend of a friend of a friend who went to a counselor.

I don’t think 2 counselors are enough for the university. I would think every faculty should have a counselor who relates to the cause not someone who studied counseling then they come to help medical students go through the stress of medicine. But I mean someone who have an experience of what they are going through per faculty, the medicine faculty, the engineering faculty, yeah, so that people are able to seek help, but then also, I believe that group engagements are very important. And I personally believe in small groups because mental health issues tend to be more personal that communal, so when we have, again am not against big groups, they have their place, and but I also think that we need to start looking at smaller groups to give people the safe environment feel, that you know, they are not opening up to the whole world and constant people actually show there is a sense of belonging. The way we have in the Christian area, the way we have care groups, these are people who stay together and they come together every day, they know one another, they got to be each other’s keepers, I think there is need for such things to start to happen per faculty per place of residence. Even the doctors that are working, I mean in every area, because it is important to share. And again, I keep saying the global village tends to connect us but divide us, because we seem to know what is happening to everyone and yet we actually don’t know much because when we come to the internet, we portray one person and then we are different people all together. So that place where you have people you can confide in is a very important thing.

But maybe also lastly, the aspect of mentorship. We tend to go through medical school, like blin people, everyone gets to bump into things and experience things afresh, and like a wise man on earth said nothing on earth is newly, but old things happen to new people. I think we lack mentorship, if there was a mentorship program, even if it meant that first year students are going to have second year people mentor them or third years por doctor this or whichever way we thought it appropriate so that when someone is in first year, they are not walking blindly, they have someone who tells them, anatomy is supposed to be this way, don’t play with biochemistry, it seems easy but it’s actually not, be more careful with this, things like those. When I was going to internal medicine in 3rd year, I met a person at the p lace where we buy fires in TASO and the person told me you are dead. Internal medicine is so hard, you are dead, and man, I went to internal medicine and by the time I realized I was not dead it was the fourth week. Up to now I keep saying I can’t go back to internal medicine so that I know internal medicine is not hard. but it was so hard for me in the first week because someone had mentioned bad about it, but what if that person had actually instilled positive energy, what if I was walking in fourth year a fifth year guiding me through, something like that. So yeah, I think we need to explore the aspect of mentorship, student by student, but also by elder people. Yeah, I think I’ve said a lot, I will pause there.

***Interviewer: Alright, thank you so much for your wonderful responses, at this moment I would like my colleague to say something, Interviewer 2, are you there?***

***Interviewer: Wow, Wow, Wow, Yeah, yes thank you very much Interviewee 6, we are enjoyed and this is very well, I think you have given really detailed information. Because surely this is not the first interview but this interview has its own uniqueness. Thank you very much.***

**Respondent:** It’s a pleasure

***Interviewer: Alright, Alright, I thank you once again, because this research is being conducted by students who are probably limited resources, we apologize for the fact that we have not been able to provide refreshment during the interview, but with hope and faith we anticipate that perhaps int eh next interviews that well share with you, probably something will be availed on your table to soothe your throat as you respond to the interview. But as per now please receive our humble apologies. Alright, Thank you so much. The meeting will end here.***

**Respondent:** Its well.
