# Supplementary material for: Perspectives on mental health services for medical students at a Ugandan medical school
Source: BMC Med Educ. 2022 Oct 25;22:734. doi: 10.1186/s12909-022-03815-8 (PMC9592876; doi:10.1186/s12909-022-03815-8)
Supplement: Supplementary file 2 — Additional file 2. [file 12909_2022_3815_MOESM2_ESM.zip › interview 7.docx]

**INTERVIEWER:** Would you please tell me about the mental health services offered at the university, the types and where students access them.

**RESPONDENT:** Mbarara University of Science and technology we have about 5 counsellors who are counseling psychologists by profession and some are placed in the dean of student’s department and others are places in the psychiatry department and others are in the faculty of medicine for the teaching purposes. So, we are placed in that way because we know the students are cutting across there are some because of the student’s welfare we handle them under the dean of student’s office. But then we are also those who could be referred because of the mental health challenges to psychiatric department for further management and we know there is a need that a counsellor is also needed that side. Not only for the students alone but also for the staff and also for the community around us. So that is the reason why we are placed in a different location because of the big need of the mental health around the campus. I do not know whether I have answered your question.

**INTERVIEWER:** Yes, thank you. I would like to know; how do students find out about these services and where they can access them.

**RESPONDENT:** Thank you. Usually when we are recruiting the students and especially at the time of orientation the university counsellor is always involved right from the word go in the orientation to make sure that each and every class is aware about the counselling services offered at Mbarara University of Science and Technology. So that every faculty and every stu dents is aware of the services being offered and where to get them. For example I do not sit in the dean of students office, I sit in the technician’s block which is the physiology department and the reason why my office is located there, strategically there are those students who have been stigmatized and they do not want to be seen going to the counsellor because when they go to the counsellor then there will be this word of saying probably you have mental challenges or you have this and that so for that reason some of them go there privately to meet the university counselor but there are also those who come voluntarily. They feel they have a challenge and they want to come. We also have another counselor who is the deputy dean of students, she is also a psychologist and a counselor in practice so she can also handle those who come direct to the dean of students for support and we also have another counselor who seats in another place where she also meets the student groups like the peer group. So for them the peer counseling is done in another location and we have another counselor with them. So mainly those are the locations where they can meet and that is how they usually do them because immediately they come immediately we tell them the information that if you want a male counselor this is the place where to find a male counselor, if you want a female counselor this is the place to find a female counselor, you want educational psychologist, this is the place or you want a counseling psychologist this is the place. Then you have the mental challenges the other one is also another place. But we can also do the referral because sometimes you aahhh find the situation have gone worse and therefore sometimes the client comes and you refer to the psychiatrists at the mental health department in the hospital. Okay back to you.

**INTERVIEWER:** Thank you very much, I have gotten that students are told where to go upon arrival in their first year, I would like to know if there is any other mechanism of communicating these different sources of mental health services to the continuing students.

**RESPONDENT:** Okay, every semester we are having different activities and for example now in the middle of the semester we normally have the students leaders training where we bring all the guild leaders, we are having around 90 guild representatives GRC members and then we also train them for peer counselling but at the same time to tell them that hey we have the counseling services and therefore if you have a problem for mental health and for any other then you can be able to meet the counseling. Then we also tell them where they are allocated, so we do not remain there. We do not stop there even the ones who are going, at the end of the semester we also have another training like the life after campus those are the things we also talk, we still train. And not only that, we train the peer counselors and those ones they they they, we normally train them to make sure that they can be able to be the point of reference for example a students can meet his fellow student in the dormitory and realize that this person need to see the counselor and probably the first aid, I may call it the first aid but the first approach this student is able to at least do some basic skills of counseling and then refer to the University counselors and at the same time we can refer to the psychiatrist depending on how big is the challenge. Back to you.

**INTERVIEWER:** So I would like to know, to what extent do you think medical students utilize these mental health services if at all they utilize them?

**RESPONDENT:** Aahhhhh I would say that ammm these service have been very very important for the students welfare because they come, they really come and attend the services and also utilize the services either from the psychiatrist I mean from the psychiatry department or from the university counselor or from the other counselors but they do, they utilize them, they have been in use.

**INTERVIEWER:** So, umm that is good to know that they have been utilized. Would you point out any facilitators that enable students to make use of these services because we know there is some stigma that can hinder people from utilizing the services, would you point out any facilitators that are in place.

**RESPONDENT:** What do you mean by facilitators

**INTERVIEWER:** Things that make it easy for students to access the services or that encourage them to take up to utilize these services.

**RESPONDENT:** What makes it easy, I think I would use the word motivator, not the facilitator, I am sorry I do not want to give you another word but I want to say what I want to tell you is the motivators, what motivates these students to use the facilities or to benefits form the counseling services. One, I would like to say that these training that we do, it also opens the awareness to the students, much as there is this stigma that you are talking about, there is also this awareness, the self-awareness, through the trainings that we have had, the students get to understand that by the way, I need the counseling services or maybe my roommate or maybe my course mate, because at times we train the class presidents and the class president may say that these students who have been missing classes for the whole of this week needs this service. And so those ones actually they led the students to bring them to counseling services, then the peer counselors that we also train. Then the peer counselors that we also train they also do the same and encourage their colleagues to come for the counseling services. And also, we have the university administrators where we normally meet them and train them, the faculty administrator can easily refer a student including the dean, they easily refer the student to the counseling service. I have had several cases where the lecturers themselves are just referring students for counseling. For example you find a student who have failed about three course units in a semester uhhh and this person is not just a dull person but probably he has some challenges somewhere and the lecturer follows up and sends this student or sometimes they give the name and we approach the student but some times they tell the student please go and see the counselor you must be having a challenge and they still come. Those are the motivator points that we use to help the students. And also, the dean of students of course he is in the middle of this because this is a student’s warfare. The dean students himself is aware of the performance because he is a top manager, he seats on the top management and he is aware of what is going through or what is going through or what is happening and even the students when they are agitating for their rights they go through the dean of students therefore he can easily identify where the counseling services are needed and we address, we focus on the direction. Thank you back to you.

**INTERVIEWER:** Thank you very much, that was good. So, I would like to know if there are barriers that could be stopping some students from utilizing these services.

**RESPONDENT:** Stigma and also counseling is a a very young profession country wide, actually in the low developed countries apart from Kenya, Kenya has gone a little bit ahead of us, but in Uganda counseling is a very young profession. But in Uganda counseling is a very young profession and so for that reason when you tell somebody that you need to see a counselor somebody thinks he is mentally sick. And therefore, telling the person you need a counselor it is a kind of saying, man or woman, this is, you are not stable in your head. So that is also a stigma attached to counseling. And also, the unawareness. Much as we are trying our best to do the self-awareness you know sensitization and doing this, but there is also a small group because the university is big, there are those who are staying outside, we can sensitize our dormitories and hostels but sometimes to some areas we have not reached and therefore you find there are not aware of the counseling services, at the same time, people thinks that everyone can do counseling and ahhhh not forgetting that there is a difference between counseling and also giving advice. Now is counseling we do not give advice, the advisors, sometimes you can approach someone to give you advice and he gives you advice. And also, the other thing is the nature of the courses. For example, the medical, let me say medical students, they are almost busy now and again, 100% busy of the time, let me not use 100% but almost 80% of their time is busy busy busy and so for that reason you find that getting them is a challenge. And also counseling is a process, by definition counseling is a process it is not one day so for that reason, if I am ahhhh, the first time I have done a rapport then the next time I want to start the counseling now the student stops at building the rapport and the next day he is not able to come back because of the nature of his course and that also hinders the counseling process to be much more effective so mainly ahhh ahhh those are the barriers, they are many but those are the few that I can remember. And also culture value, let me not escape the cultural values, the culture is also another barrier for counseling because we are coming from.. we have the different culture and so the culture itself is also another big hinderance of counseling services and then the belief if you are.. if the counselor is a Muslim sometimes you say ahh ahh ahh this is a Muslim I am not going there, you forget that this s a professional service that somebody is going to give and you say maybe ahha ahhahh this is a born again, he is going to preach to me about this. So, the belief, the culture and yeah, those are also added onto the barriers of counseling. Back to you.

**INTERVIEWER:** Thank you very much. I would like to know if there is anything being done to counter these barriers of people not knowing about the services and also the cultural barrier. Are there some things being done to counter these?

**RESPONDENT:** Okay ahhhh, the interventions that we are doing one is ahhhh of course the university is employing the counselors from different religions ahhhh there are those who are coming from the Pentecostal background, there are those who are coming form the Anglican background, the catholic background. You know that also breaks, because if somebody comes and I just ask as I build a rapport, are you comfortable with me or you want somebody else we have this person we have this person, somebody will just say yes, I need this, Maybe I need a female or maybe I need a male. That is also breaking the stigma about that and I mean the intervention we have done. And also, sensitization has been done almost every semester ahhh to make sure that ahhh we break the hinderances. So the sensitization has been done severely, if it wasn’t for COVID now we would want to almost come to every class to make sure that we are breaking that barrier. Ahhhhh mainly those are the interventions that we ar21 e doing to make sure that we are breaking the barriers. So having different counselors and also sensitization and yeah, and of course there are others that have been made by themselves that is like counseling courses have been introduced in different universities so therefore counseling profession is no longer an issue because Kyambogo has been doing it and they have gone to another, UCU have been doing it up to the masters level aahhhh of course Makerere is already now on PhD and postgraduates, Bishop Stuart the same and other universities are doing the same to train more counselors and therefore that is no longer ahhh much as there are still few in circulation but that is ahhh ahhh being handled at another level.

**INTERVIEWER:** Okay, thank you. I would like to know if there are mechanisms of evaluating these services that are being utilized by the students.

**RESPONDENT:** Okay now, one is that whenever we do these trainings sometimes, we give them the papers to give us the feedback. And the reason why we are giving them is to see whether we are effectively helping them or we are not helping them, so we have been doing it almost every semester to give them the papers and also, they give us the feedback. But also, in another way ahhh ahh is what we do is ahh we have the peer groups that are also handling counseling, they also meet them. Our students meet them and also when they meet them, they discuss and find out how best we can support them in terms of the ahhhh as far as the counseling services are concerned and then we meet and discuss. And of course as a department we also seat and evaluate ourselves because we have the workplan and we set the goals at the beginning of the semester, so at the end of the semester we evaluate ourselves and see if we have effectively ahhh utilized our work plan to reach the students if not, then of course we can score ourselves and know yes we have done but we have failed at this level, for example like these two semesters almost we know that we have somehow failed to reach our target because of the COVID and these breaks that we are getting. Before we handle this then there is a lock down so, those are the ways that we normally evaluate ourselves, back to you.

**INTERVIEWER:** Thank you, that is ahhh very insightful. So, what would you recommend to be done to improve these services at the university?

**RESPONDENT:** ammm, at the moment I I aaahhh as the University counselor, I am of course I have already shared it with the management and it is in plan we want to do a counseling, ahhh a big counseling center where the students can come and relax, so in our future plan at Kihumuro campus we are also planning to have the counseling center where the student can come and relax and sit, and you know and ahhhh you known other universities have gone up to that level but Mbarara University but of course as some people who have been struggling to go to the new campus we are now looking at how best we can expand and make a counseling center where we can ahhh the children counseling room because we should be having where we can counsel the children of our staff and also the students and different ahhh departments within the counseling unit so we are working on that and that is our goal where we are planning to go, to make sure that we have a counseling center and everyone can come not only to be counseled but you can come to relax as a student when you are in the pressure of the course work. Back to you.

**INTERVIEWER:** Okay, thank you So lastly, I would like to know if you have any recommendations to ensure utilization of mental health services in the University by medical students.

**RESPONDENT:** Ammmm One of my recommendations have been that of course there are few lecturers who normally do the follow up of the academic performance of our students, but there are also those lectuers sometimes who does not mind about the performance, the decline or the unsual decline of the students to make sure that they refer them for the psychologist, for the counseling psychologists to support them. If these students have been performing fairly well and all over a sudden there is a serious decline, then it should be a collective effort of every lecturer to refer this student and not only to refer but also to talk to this student and probably you bring this student to the know of the dean of student’s department and then we can be able to support them, but there are those who dose it. I am glad that in the faculty of medicine there are those who does t but there are those but there are those who does not care about the performance, it declines and some people keeps quite but we have always been encouraging every staff that when you see the performance is not doing, is not going in the right direction, then let the dean of students department be aware so that we can immediately kick in and support this student because I believe that every student who comes at Mbarara University of science and technology is capable to go back with his degree irrespective of what. But he should be able to go back with his degree because he has come with his marks and his right qualifications to do the need, to do his dream and realize the dream. Back to you.

**INTERVIEWER:** Alright, so basically this is what I wanted to find out from you, ahhhh unless if you have any last words, I would like to thank you and conclude the session.

**RESPONDENT:** Okay, thank you so much for the interest in taking this research and I with you the best, this is [mentions name] the university counselor, bye for now.

**INTERVIEWER:** Okay, have a good night. Thank you.
