# Supplementary material for: Perspectives on mental health services for medical students at a Ugandan medical school
Source: BMC Med Educ. 2022 Oct 25;22:734. doi: 10.1186/s12909-022-03815-8 (PMC9592876; doi:10.1186/s12909-022-03815-8)
Supplement: Supplementary file 2 — Additional file 2. [file 12909_2022_3815_MOESM2_ESM.zip › interview 4.docx]

**DEAN FACULTY OF MEDICINE**

**Interviewer:** Thank you very much for accepting to take part in this study. I would like to know more about your role in your years of service at the university towards the mental health of students as well as the utilization of mental health services by medical students.

**Respondent:** I hope I have gotten your question right. I have been involved in the care of mental health clients not as a student only, but as an intern , post graduate student and now a specialist. Now as an obstetrician, I come face to face with some cases of mental health especially postpartum psychosis, depression. That is as far as my active involvement in mental health management can go but I’ve gotten involved with students that get some spikes of mental health challenges.

**Interviewer:** You have said that there are students who get some mental health challenges here at the university, I would like to know basing on your experience with these students, what mental health services are in place to help these students to cope with the mental ill health?

**Respondent:** We have counselling services which are offered by the university counsellor. We also have a counsellor in the faculty of medicine and services available in the department of psychiatry where attendance to these cases is by psychiatrists and psychiatric officers.

**Interviewer:** You have both counselling and psychiatry services. How do these students get to know about these services?

**Respondent:** Usually they get to know about them when they are reporting to campus during orientation. I know that the office of dean students usually speaks about the services available to students. When we have some specialists invited to give some brief talks, they hint about availability of some of these services. I know that students when they are rotating through department of psychiatry they get to know about services offered there. Another way that students get to know is peer to peer. I know of students who have said they got to know about services from their colleagues. Those are some of the ones I recall.

**Interviewer:** Are there other ways you have got to know how medical students cope with mental ill health without utilizing these mental health services?

**Respondent:** I know there is peer to peer support. I have quite often listened to students trying to support their fellow students who have got some mental health challenges. I have received students coming to my office telling me of how they are trying to help their colleagues to access lectures and pull through some of the challenges they are facing.

**Interviewer:** You have talked about students getting clinical management from the psychiatry department which doesn’t only offer care to the students but the entire community. Do the students receive a customized care by virtue of the fact that they are from Mbarara University?

**Respondent:** As far as I know, there is definitely no special clinics for only students, it’s a general clinic. However the students that go there are given a priority from the fact that they are part of the clinical delivery pathway and receive some degree of preferential treatment, I have interacted with my colleagues and students as they are receiving care from the psychiatric department.

**Interviewer:** You have talked about the services offered. What do you think are the factors that limit students from utilizing these services? Do you think that these services are fully utilized by the students in the faculty of medicine?

**Respondent:** I think there is a gap. I wouldn’t say the utilization is optimal. Some of the challenges include lack of adequate private space where these students could tell their stories and get attended to and then, there are students who fear to follow the normal clinical pathway because they will be found out by their colleagues. Thirdly, some stigma may be attached to that. We do not have adequate numbers of counsellors to attend to every student who has got a mental health problem. Those three factors would answer the question.

**Interviewer:** You have said that the fact that there is some stigma, lack of personnel to offer the services as well as lack of private space for students to be free are the barriers to utilization of mental health services. Given your current position in the faculty, are there efforts that have been put forward in order solve these barriers and make mental health care better for medical students?

**Respondent:** Well to start with, we are trying to identify specific office space for our counsellor in the faculty. We have had some engagement with the psychiatry department and dean of students’ office to join efforts and try to identify students who have these challenges. We have and continue to interact with the student leadership, not just the guild but we are beginning to interact with MBUMSA and other program associations.

**Interviewer:** You have talked about the interaction with the dean of students’ office and we have been informed that they also have a counsellor. Is there a liaison between your office and dean of students’ office to ensure that all these come into place?

**Respondent:** Yes, you are right. We are putting efforts to ensure that all the relevant stake holders are involved right from the start as these students are identified. We know that as they receive treatment they need the support which cannot only be clinical but peer support too, that’s why we are trying to involve the student leaders and students themselves. One thing that we have not worked on vigilantly is the need to let the community know that these challenges exist and people should not be surprised when students come up with such mental health issues.

**Interviewer:** You gave a range of services which students can use to cope with their ill mental health which means mental health is being prioritized in the faculty nowadays for medical students. Is there a form of evaluation towards these services to ensure that there is continuous improvement and that students get the best experience as they access them?

**Respondent:** I am not sure about any evaluation. I might need to interact with the dean of student’s office and the department of psychiatry about that. I have not come across any programmatic evaluation of these services.

**Interviewer:** At one point you said one of the ways the students get to know about these services is through their orientation week, I would like to know if you think that is sufficient, are there other ways the faculty is trying to ensure that these students get to know about these services? We believe that some students don’t attend orientation and some while there, are not attentive enough.

**Respondent:** I agree there is need for continual communication and sensitization of the students in the different years. You are right to say that some students don’t even attend orientation and they are quite a number. Meaning that as they come it is possible that we can organize with the student leadership to have some sensitization opportunities whether it is a mental health week or brochures. There is also need to sensitize the faculty so that once in a while as we interact with the students, there is some precise information we can share with the students even in our lectures, the compound and many other avenues.

**Interviewer:** Do you have any recommendations towards the utilization of mental health services as well as the mental health situation in the university?

**Respondent:** We need to identify enough numbers of people who can offer the service. We need to have adequate space where the services can be accessed. We need to involve the students themselves in coming up with possible solutions to challenges that affect them. We need to have a program for continued sensitization through either physical or virtual or whichever means. We need to identify resources to support the provision of those services because quite often students may get prescriptions that are not readily available in hospital and need to find ways of accessing them

**Interviewer:** Thank you very much for accepting to participate in this interview.
