# Supplementary material for: Perspectives on mental health services for medical students at a Ugandan medical school
Source: BMC Med Educ. 2022 Oct 25;22:734. doi: 10.1186/s12909-022-03815-8 (PMC9592876; doi:10.1186/s12909-022-03815-8)
Supplement: Supplementary file 2 — Additional file 2. [file 12909_2022_3815_MOESM2_ESM.zip › interview 6.docx]

Transcription by David 1.1

**INTERVIEWER:** I would like you to tell me about the mental health services offered at the university, in terms of the services which are offered and how students access them.

**RESPONDENT:** We have both formal and informal structures through which an individual can access mental health services. Aaaaah the basic level we have peer counseling services and these are through student to student counseling. We have several peers educators groups that are trained in basic counseling and these provide one on one counseling to students that require some level of support. For example, if someone is having a challenge with say the balance of their academic activities or making decisions about relationships, then we believe that through training the peer educators, the interaction they have with fellow students is a bit more guided and informed towards a helping relationship. And as you very well know that mental health services are on a continuum from just access information all the way to treating illness, so we believe the starting point is through peer to peer interactions and eventually counseling. Then the other level that we have is where we have the university counselor, we have particular individuals that are appointed for that role. And the university has three counselors that are appointed. I am assuring you are aware of them [mentions names] one plays a double role of counsellor and lecturer. But in addition to that, some of our staff members have a background either by their academic training or by their work experience to supplement the role of the counsellors especially the dean of students department so we do provide that particular level of support with in the university structures of course and the university mandate. The third level which is now more close to the treatment of either mental illness or if someone is having a mental breakdown and close to that part of the continuum we work with the psychiatry department where we link the students to psychiatry care if they need it, or to some level of psychotherapy that is beyond the counselors mandate. So those are the mental health services that we have.

**INTERVIEWER:** Thank you very much. I would like to know. How do students find out about the peers and how do they access the counsellors?

**RESPONDENT:** Usually.. we have two, we have three peer education groups right now. We have the MUST peer project, HIP roar peer project, then we have the MSF which is medical students for choice. These particular three groups have contacts in terms of their leaders and some of them have physical structures where students can walk in. In addition to that the students reach out to fellow students with in the hostels and we try as much as possible to enroll students from various programs in the university so that they are the contact for those particular programs. Many times, we have had students come in and say we have a colleague who is not attending class, that is simply because the peer educator or counsellor will be able to identify those particular issues with in their communities. So, in terms of contact as you asked, it is, there is a physical address where they can go to, but also the peer counsellors reach out to their fellow students through the different opportunities and interactions.

**INTERVIEWER:** Would you say that students utilize these services?

**RESPONDENT:** Yes, they do. As someone who is directly in charge of that docket, the peer education docket I clearly see the way it comes to, if I can call it fruition. Many times we get informal reports about a student showing signs of a break down, a nervous breakdown or anxiety and then we contact the students as the dean of students office, so many times the information that comes through from the student community is either through the guild leaders or the peer educators most of the time. So we believe the services have been utilized because we are able to tract students, we are able to know this student has not been attending lectures and then you find out probably they had a break down, in cases of rape we find out that this student has experiences say an attack or has been raped and we reach out to the student before it is too late because many times the student community does not know where to go but the peer counselors and the guild leaders know where they can direct the students so the services are utilized, of course we would like them to be utilized more, however, we would need to spread out the program and maybe train more peer counselors and peer educators.

**INTERVIEWER:** So, I would like to know in regards to medical students in particular, do they utilize these services.

**RESPONDENT:** Pardon?

**INTERVIEWER:** How is the utilization of these services by medical students?

**RESPONDENT:** Some of the counsellors are medical students and they do utilize the services. Like I said, normally it is aahhh, if I refer to, if I can create an analogy it is like a tree, a vine. For instance, the time when we had a student have a nervous breakdown, the student did not come to us and that was a medical student actually they could be more, they are about two. So, we found out from the medical student leaders who belonged to MSF that we have this student who has a break down and they are at a particular restaurant and then we went into action to pick up the student. Another one the student was still in the hostel but they were incongruent in their interaction with others. And still it was a medical student who linked us to the student who had challenges. So, the medical students do. However, I know why you are asking that. I should emphasize that we need to have more peer education or peer counselling services however the gap can be bridged by having more students equipped in counseling skills such that they are able to be the link between the students and the university officials. So, for medical students there are few counsellors in comparison to the number with in those various programs but the few are actually doing come work.

**INTERVIEWER:** So, I would like to know if there are things in place which make it easy for students to access these mental health services. Things that facilitate the utilization of the service.

**RESPONDENT:** So, the easiest place is the dean of student’s office. That is where we say formal mental health support can be accessed. Because we be a link to the counsellors, we are a link to psychiatric care or mental health care. However, there is a designated office for the university counselor and it is not with in the department and it is not with in a setting that students will be looking at who is moving in. It is, I do not know if you know where the office is. But its location is ideal because it provides some level of confidentiality both for the department. Because if you come for counseling services from the university counsellor, the other university staff do not have to know your business especially in the dean of student’s department. So the office is located with in office/student accessible premises so that when you move to that office you do not ahh you are not standing out as someone who is seeking aahh counseling services you could be going to meet fellow students or a lecturer or a laboratory staff, that particular structure has many people that access it and so the counseling office is with in the structure for a reason, for confidentiality.

**INTERVIEWER:** I would like to know, are student communicated to or reminded about these services because some people may not be knowing where to go, is there a mechanism in place to publicize the services and encourage students to seek help.

**RESPONDENT:** So we have the orientation program, the first time we contact students once they join the university we actually do have a session where we talk about student support services and this ranges from hostel access and also including the counseling component where they can access counseling services and we even point out some of the challenges that may face that may cause them to benefit from these service for instance academic pressures, relationships, family problems and things like that. So that is the first time, the very first week when new students are reporting, during the orientation period they are given information on where to access these services and we introduce all the counsellors and all members who are able to provide counseling services with in the institution. Two, the faculty with in all the programs do normally direct students towards our services ammm either by identifying students who could benefit from the service. For instance we have had moments when the lecturers or the administrators with in the faculty will write and direct or demand that the students come to our offices because of what they have observed with in the student so those are the major ways with in which the services are known or are popularized if I can use that word in addition to having information on the university website and the dean of students office. However, I believe we could do more. For instance, have probably running adds now that it is an active social media error where messages can be sent to various contacts of students, I think we can do more in that regard, yeah in terms of popularizing the services.

**INTERVIEWER:** I would like to know, in your view are there things that could be barriers stopping students from coming for these services.

**RESPONDENT:** Yeah sure, ammm there are various reasons. Right on top of my head, there is ahhh, I think that most times we have a culture where talking to a stranger is not something that is commonly done in the African societies especially if you come form different cultural back grounds for example the bantu the Baganda the Banyankole, I am not going to talk about my problems with a new person. That I think for me is a very big influence in regards to accessing mental health services in addition to the stigma once you have moved from just feeling the pressures of life whether the pressures are from academia or social or family and the problem gets worse and move towards mental illness, okay, then the stigma associated with that may ahhh prevent somebody from coming to access these services. Many times, students come in and you realize that this student has come in requesting from supplementaries however they have several say they have experienced a traumatic incidence say they are not able to deal with lose of a loved one. So, they are coming in to ask for say support regards to accessing special exams. Ummm but in interacting with the student you realize this student maybe dealing with this lose and probably they are slipping into depression. The moment you suggest that you need to see the university counsellor and you need to be supported and be able to cope with the lose ahhh before you even try to concentrate with the exam alone, the student feels you are telling them they are mentally unwell, they feel a certain level of stigma. So that is the other reason. Ammm and then the other which could be, the ones I have mentioned are more of personal reasons based on how someone feels, however with in the structure I think we could benefit from having more counsellors with in the institution possibly by faculty so that it is more accessible as a service because right now three counsellors to a ratio of over 3000 students is really thin. And then the other reason why these services may not be utilized in the fact that the students, I do not know if I should say the students but in terms of university there is a way the environment makes us believe we are independent and therefore when individuals are having challenges there is a way they feel like these are my problems, I do not know if you understand my point. So when some says when someone is having a challenge say if you come from secondary school and you already have challenges say with anxiety with depression or you have some level of ADHD or you have some autism and it interacts with your social capabilities so when they come in they hope to continue to receive the mental health support from where they were receiving it from say at home or with in the neighboring facilities in their locations and they forget that when you are in the university the other programs will run as though they are normal for everybody yet you want to constantly travel back home every weekend. And therefore the person will try to insist to keep their problems to themselves if I can call it that so they will face very many challenges but the stem of the challenge will be why they would not access our services is because they believe that is their problem, they have had it and it is theirs alone. So that is the other reason I think students would not access our services.

**Interviewer:**  Would you say there is something being done to counter these challenges.

**Respondent:** Well, with the person challenges I mentioned, the stigma the ahhhh this is my, felling like you are too independent to seek any help which other challenge did I mention, personal challenge did I mention. Those personal challenges we keep talking to the student community and we keep encouraging those lecturers to try as much as possible to ahhh encourage students to come to our offices or even provide the counselling themselves. Regarding the formal or the structural challenge. We have contributed as a department to the writing of the strategic plan of the institution and we particularly emphasized the need for a wellness center for the students community because of the nature of the academic load especially for the postgraduate students most especially for the medical fraternity because for instance when you look at the COVID times the students are going to want to continue practicing or looking for places where they can be locums however the threats that come along with that responsibility are high, the risks are high so the stress and everything is high and we need to have a wellness center that responds to the needs of the students so we clearly wrote down a plan and emphasized that we need a wellness center to cater for the psychosocial health of the students community. We are aware that the medical students have challenges that may requires special attention so that is why we suggested that. Maybe the other thing is that we have encouraged most of our staff ahhh especially in the dean of students office because that is where the mandate to provide mental health support and psychosocial support, we have encouraged and funded staff to get training in counselling and so far two of our staff have, the two are in Kihumuro campus now so we hope that more will undertake the counselling course that is offered at a higher level and certificate level which is affordable for the institution to fund.

**Interviewer:** Thank you, I would like to know, is there any other thing that you think is not being done but you think should be done to counter the challenges/ barriers of accessing the mental health services.

**Respondent:** Is there any other?

**Interviewer:** Any other thing that you would recommend to be done to counter these barriers.

**Respondent:** Well, I think I have mentioned most of the things that come to mind to improve the mental health services that come to mind to improve the mental health services as I spoke about the existing services and the challenges. So really for me it is really strengthen the peer education services and counselling services so that we have various representatives from the different programs and then have various ways to popularize the services that are existing, then the other thing is have more staff trained in mental health support services say counselling for instance and also set up a wellness center where individuals know this is where they are going to go and have a contact from the wellness center under each faculty.

**Interviewer:** Alright thank you very much, so, I would like to know if there is a mechanism for evaluating these services to find out if they are good enough if the students the students are satisfied with them?

**Respondent:** Ahhhhh I am not sure, probably the counsellor will talk about that. Most of the time the service utilization can only evaluated with how many people came to the office, the counsellor may have a way to find out whether the relationship is helpful and when people come. Because of the nature of the services it is hard to really quantify or qualify if it is good enough or terrible, if I can use that word, so we do not have a particular say fully fledged mechanism to evaluate the service, we have our usual way that we use for all services for example having a summary statistic of how many students have come this year and in what general area where the student challenges so that when we are addressing students the students we point out those particular areas for instance now drug abuse has been quite on the high and that information comes from the counselling department so we pick out general areas however having say like a qualitative assessment to find out how the services has been helpful is not something that we do often. So unless my colleagues essentially my supervisors who are involved in the evaluation process have another mechanism that is utilized form the top of my head I know we normally discuss at the department to identify the common challenges whether it is betting, at some point we had betting as common challenge that would merit people to access mental health services so that they do not go into the depths of suicidal depression now it is more of drug abuse. Now recently it is more of drug abuse, so we have ways of picking out information from the counselling community to find ways of improving or responding to the needs of the community. However, I think that researches like this will provide a good a good feature becomes it comes from the general public however is may not be as specific to the counselling department, it may be generally in terms of where we get mental health support.

**Interviewer:** Alright, I think basically this is what we wanted to find out from you and in conclusion, you mentioned a couple of things that can be done to improve the services maybe you can just highlight that one may thing which you would recommend to ensure that medical students utilize the mental health services so that we can have more students utilizing these services.

**Respondent:** Medical students in particular?

**INTERVIEWER:** Yes, medical students in particular.

**RESPONDENT:** Well I think it all goes back to providing a place where it is identifiable to the students but one also where individuals are aware of the specific needs or the special needs of the medical students. So it could come in form of a person to contact or if all resources are available it could come in form of a wellness center. We have seen that institutions from the first world normally have like a wellness center or a counselling unit where each students regardless of whether you need the service or not you come in with for your, if I can call it a semester appointment with the psychologist or with the counselor so that they are able to know this service is available and this is the person to approach if you need it but before you do or incase you think you don’t we still have to interact with you. I know some institutions that have some level of medical education where you have an appointment each semester or each year with the counselor or psychologist to just interact with them as so they know or they can just have an interaction with you to see where you are at as an individual not because you have any challenges in particular but you just have to .. they just want to now you you are and why the service exists. With that in mind, because that is the top notch service, with that in mind you have to think of how many people you need, where do you need to set up, in an ideal situation that is what will be required but in our setting even having an individual that comes or is appointed to the faculty to the undergraduates and maybe mostly for the people who are with in providing healthcare either as students in training or as individuals who are working would be very beneficial to the medical ..ahhh ahhh students and eventually to the faculty.

**INTERVIEWER:** Alright thank you very much. Basically, this is what we wanted to find out from you. Have a nice day.

R. Okay bye.
