# Supplementary material for: Perspectives on mental health services for medical students at a Ugandan medical school
Source: BMC Med Educ. 2022 Oct 25;22:734. doi: 10.1186/s12909-022-03815-8 (PMC9592876; doi:10.1186/s12909-022-03815-8)
Supplement: Supplementary file 2 — Additional file 2. [file 12909_2022_3815_MOESM2_ESM.zip › interview 5.docx]

**Interviewer** 1: Tell me a bit about yourself, the position you hold in the university and any few interfaces you have had with mental health issues and the services offered.

**Respondent**: I serve a population of about 4500 students. Ahmm… the university is a multi-disciplinary one having different programs from different years both undergraduate to post graduate and even certificate courses. Ahmm…the distribution of the population is ahmm… Faculty of Medicine, Faculty of Business, Faculty of Science ahmm..Faculty of Applied Sciences and Technology, Faculty of Computing and Informatics, Faculty of Interdisciplinary Studies being the least.

Right from the time I joined the university, I have served in different capacities of student assistance and there have been several…several experiences of students facing challenges of mental health. To start with ahmm a good number of students who come to university, MUST inclusive, one; are not doing the programs they want to do and there’s already that experience. Am I answering the right question?

**Interviewer** 1: Yes, you are. Just go ahead. Feel free

**Respondent**: So already ahmm. Already have that sort of ahmmm depression and sadness that comes from not getting that program they wanted or let alone that coming to the university they wanted so the way they are treated when they come into the university really impacts how they are going to continue their life at the university. So before they are even influenced by the environment that the university comes with, there is that…there is that ahmm sadness that comes with that. Let alone that, the fact that…even while many people assume that ahmm..being in South Western Uganda, a good number of people hail from the district itself, that is not the case, very many people are coming from far places across the country and we even have international students who are getting the culture shock just from being in a different sort of setting than they were raised in. And equally, aahh.. just coming from high school to university, that’s a completely different environment that I feel very many students are not prepared for…ahmm.. it is just an expectation that you will be fine but it’s really, it’s like how a child come out of the womb and comes into the physical world, there’s that shock and they have to be soothed and made comfortable which we don’t really get, which I feel we get but it is not tailor made for the students themselves. There’s that impression of you’re strong, the the little motivational speech I’ll give is just sufficient to give you the boost you need yet this kind of support must be continuous all through your campus life.

So, I will not assume that students come with different vices where they came from but there’s that possibility. I have several experiences of students abusing substances, taking substances and from the ones that I have heard, I had a student who…one had an overdose of students and this student who had an overdose, this was his first experience taking drugs and the one who happened to give him the drugs had been taking drugs for about 5 years and this was a first year. For the one who had been taking drugs for 5 years, his first experience was obviously was back in high school and then this one due to making social networks, being friends with this person, had an experience of substance abuse and this is quite common.

There are hostels that are widely known among the students and I have equally come to realize among the top management that they are bases of substance abuse. Due to different things ahmm…in, I think in my first year and second year, I have been a sexual and reproductive health enthusiast, peer educator and we have had experiences where very many students engage in sexual activities, some of them are not consensual, some of them eventually need emergency contraceptives and even the way they come out to ask you is filled with a lot of distress and there’s no specific place besides the students peer educators that they can comfortably go to and get the assistance they need. And there are those that go on to have abortions which is illegal in Uganda but we know it happens and we… support them in in when they have gone through the experience and they come to you for help, you can not turn them away, you’re basically the first person they have come to so you have to act rationally and be helpful to them.

Even being a leader in those different spaces, it is quite traumatic for the leaders themselves because you’re constantly being exposed to the horrors that different people are going through and therefore it is hard to keep a straight face, it is hard to alienate yourself from these experiences and it even takes you back to your own past, your own history, your own family and relatives, it really a tough thing to handle but you have to as a leader, you literally don’t really have a choice. So yes, there are definitely countless mental health ahmm..issues in the university in and around the university that are not being dealt with. Ahmm… I believe ahmm..the services that we have at the university include having counsellors, we only have one official counsellor, and then the other people are basically posing as counsellors. Ideally, in retrospect we are all counsellors, we can all offer counselling services but there’s a difference with someone who has been trained because counselling is not something you are born with, it is a skill and you can learn and can be trained to do it perfectly.

So, we have one counsellor and then we are also told that you can go to the warden, now the deputy dean or you can go to any lecturer who you feel you can talk to. But while we can do that, it is basically because the structure to offer counselling, is inadequate, it is insufficient so I can definitely say that we do not have skilled, trained human resource to deal with counselling. Let alone that, ahmm yes as a university that has a medical school, we are attached to a referral hospital, we can benefit in one way or another from the services being offered there. However, I can tell you that having had the position that I do, there’s an illusion that there’s ahmm..a direct contract between the university and the hospital to offer services to the students but I have come to realize that it is more like a gentleman’s handshake, “oh this is a MUST student, they are our neighbors, let us help them.”

So even when someone in the hospital doesn’t help, they’ll not be accountable. There’s a long story behind that but this means that very many students especially outside the faculty of medicine, leave alone that outside the Bachelor of Medicine who directly interface with the personnel in the hospital usually don’t get the services they need. So you find a program like MLS or Pharmacy, they don’t really practice with the hospital or with the doctors so they are not really known. Even they at times don’t get the services they need in the hospital so if that is happening within that faculty just imagine what other students in other faculties are going through. So if I am to make a rough estimate that’s just less than a third of the entire university population. So there’s that challenge as well. As it makes it really hard to access these services so the stigma around mental health in itself is not rally solved or improved by all these different infrastructural challenges.

**Interviewer** 1: Okay, Thank you for that. Ahmm I guess we have gotten idea about the type of mental health services offered but ahmm maybe what I have not understood is for those students actually use these services because you hinted that some use them, how do they get to know these services?

**Respondent**: When the students come into the university in the first year, they…all of them are oriented, they must attend an orientation process where different personnel in the university, different relevant people in the university come and ahmm..explain to them the different services offered. I realized ahmm, really most, while that service is offered, the attendance is quite low, most most first years do not attend that program but it is there and the service, the information is relayed to them. Then ahmm…even all through ahh…the different associations that work for example when I worked, when I I actively worked as an SRH peer educator, we were working closely with the Dean Of Student’s office and We were made aware, we had trainings as as ahmm.. leaders and were closer to the the administrators and got to know what was being offered so that we could relay it to the students. So, there’s also that arm where through the different outreaches and programs, ahhh, students can relay to fellow students that these services are possible. Then, equally, through the university website which unfortunately, very many students do not, majority of the students do not really go there to just look through and see what is being offered. There’s also a section where these different leads in those areas are are presented to the students. Even without that, there’s a Dean of students office that deals with every single student’s issue. If a student is unaware of where to go. They should go to that office and they will be redirected.

And also through different student leadership including the guild, this information Is relayed to all guild members, this includes leaders from each program and faculty so all these people are aware of this information are at the grass roots level and can relay this information to the students themselves. This means that in every small constituency, whether it is a class, whether it is a program there’s someone that aware of it. Even when they are not, those student leaders are aware of other leaders within the university and can inquire and then relay that information to the rest of the students.

**Interviewer**: So if I have a mental health problem I have to go through a student leader, noted. So personally, have you used these services?

**Respondent**: That’s not the only way you can get help. But that is the most clear-cut way you can get help. Because if you’re ill, you don’t need a student leader to go the hospital or nurse. But if you are unaware of where to get the service, you can ask and get directed. Then once you are aware of that structure, fellow students can be in position to relay that information to those that do not have it. So that’s an official, decentralized structure but the service can reach out to everyone in their different places.

**Interviewer**: So, if I am first year who did not attend orientation who may not who my student leaders are and I am not aware of the student website, what is the way forward for me?

**Respondent**: Definitely, that first year, you are going to face a lot of challenges. The problem with mental health is that while health seeking behavior all across the board is pretty low, I must say for mental health issues is even lower and cannot easily be identified by people around you. The issue with mental health is that most of the time, even the sick person themselves do not recognize that they are ill. And being a 1^st^ year who has left their usual community and come to a new place, you may not even have close friends, who may identify the change in behavior to see how you can get help. So all those different things as a first year make you more vulnerable and even hard to identify as someone who needs help.

So, what we can do for this first year is give them a supporting environment, that if you have any issue whatsoever, kindly reach out to these people. If you are ill, we try to just make them embrace the fact that if you are facing any challenge, you can reach out to these particular people for anything and they will be of help. So even if you have missed the first training, we normally advise that if you can’t reach out to the counsellor, you are free to reach out to anyone, including your lecturers, the faculty staff, the faculty lecturers really ahh…classmates or course mates try to give you an enabling environment even while for example there are counsellors, some people prefer to talk to people who they know, who they can confide in, who they can trust. So ahmm. These students should utilize people around them Even if the leader doesn’t know how to get help, they can inquire. That same model of inquiry works for the first year, they can reach out and get help.

For the first years who I have worked with on their mental health challenges, it is usually another person who calls me and tells me that there’s a problem with this person, come and help. So it really comes down to the community around you because majority of the time even I myself as a person when I am feeling sad or depressed, it is hard for me to come out and tell even my close friend that I am sad and depressed, usually, they have to sort and see that there’s something not right and maybe guide me into being aware that by the wat you’re acting different, you seem low, you seem sad but even while you’re going through the motion, you feel like I am strong enough, I will deal with it. So if I had a mental health challenge, even with all that I know, I know it is not going to be easy for me to call the counsellor whose contact I have, I will prefer maybe to let’s say even call you, I may even call you. So, the the structure of getting mental health services, it is almost just for the university to tick that expectation but usually, I believe, it is the support system that offers that first ais, you know, they give you that important first assistance that can enable you to live long enough to get the official you know, more skilled form of mental health service.

**Interviewer** 1: So you hinted on it a bit, I wanted to ask if personally you have used some of the mental health services offered by the university.

**Respondent**: Yes, I have. Yes, I have. Ahmm…

**Interviewer**: Why did you use them?

**Respondent**: Ahmm..probably ahmm..at the time I know I have talked to different ahmm..people available in the university when I am facing a challenge, when I am maybe unsure of something, when I am not feeling well, like you’re sad, or I am just not feeling my best you know and you feel like, it is getting to the point where it is affecting your productivity, your well-being, it is affecting your motivation, you know. You find yourself, the times I have I find that I am withdrawing, I am not enjoying the things that I used to, I am letting go of things that in the past I found quite fulfilling. So, when I do notice that problem, I try and get help. Usually, I have never spoken to the official counsellor but usually I speak to someone that I look up to, someone I feel is invested in my life, in my well-being and will be compelled and committed to assisting me. Those are the situations when I have utilized a mental health service within the university.

**Interviewer**: And so that person that person that you talked to, are they in the official infrastructure of mental services in the university. If I had a problem is it someone I could approach? Or is it more of a personal interaction?

**Respondent**: Yes, ahmm… they are in the one of the people I have interfaced with is in the official infrastructure of mental health, they work in the Dean of Student’s office and the Dean of Student’s Office deals with all these issues including welfare and mental health. Every issue that is not really academic though they also deal with academic issue, the Dean of Student’s Office deals with it; when you lose a parent, when you…even applying for a dead year, whatever your reasons are, you have to go through their office. So that office, even the mental health services are under that office, all health issues are under that office. So yes, you can definitely reach out to that very person and get help from them.

**Interviewer**: So the times you have used these services, have you been satisfied with the services offered? Or do you have any concerns as regards these services especially when MUST students use them?

**Respondent**: Probably, my issue would be follow-up. Following up when someone comes to you for, in my experience, maybe I come off as being self-sufficient, I don’t know, but ahh…for following up when someone comes to you and they’re getting help, staying committed to that person and finding out if they’re really okay, if they have relapsed or if they need more help. That’s the problem, I don’t think referral is not as widely used. Most people are not trained really in counselling, they think they can help you. So…though also mental health is not something you can easily just refer because you are dealing with someone’s confidential issues, you’re not sure if they want to go somewhere else. So maybe I can understand why people try to help you by themselves but I feel we should really work with other especially more qualified people to see that we help the students that come to us.

**Interviewer**: Okay ahmm also, given that you interface with a number of students, do you get to find out any challenges they face when they’re trying to get to these people, when trying to access these services or even in the process of ahmm… using these services. Are there any specific challenges they face?

**Respondent**: Uhh.. The first thing most especially like now for substance abuse, being a crime, most students really fear to get help from the university. Ahmm…not knowing that by virtue of being a university student, as long as you go through the university process, there’s a certain form of protection that the university will try to give you until they absolutely can’t. Even though you’re in a crime, unless you’re absolutely guilty, there’s a certain form of protection the university will give you. So majority of the student for those who are engaged in criminal activity will fear to actually get help from the university. And then there are situations where the university will have to declare if it is criminal activity to the police or to the security team what is happening. Then like I said, the issue of the number of qualified personnel to the n umber of the whole population, it is quite large. It is roughly 1 to 4000 ratio but now that we are putting onboard other support stuff, it is still quite insufficient and because other people that are supporting the counsellor are not equipped or responsible for mental health, it is not their…they cannot be held accountable if they do not help someone. So that in itself can really discourage students from getting help. Yeah and also the stigma involved with it, most people would prefer that their situations or their issues remain private so that also really discourages getting help. Yeah.

**Interviewer**: And the other side of the coin, what do you think facilitates and makes it easier for the students or made it easy for yourself to actually access these mental health services?

**Respondent**: Having a relationship, you know having a personal relationship with some of these people, it is easier for someone when you know and trust them, it is easier for you to confide in them. The very people who I go to, usually, I have had a personal experience with them in other things, so there’s a certain friendship that has formed so it is easy for me to go to them. Maybe, if I had a very terrible experience I would be compelled to go to someone who I don’t have a personal experience with. But I know others who would prefer to go to someone who they don’t have a personal relationship with so they can open up completely. So in my experience that’s what encouraged me to seek help.

**Interviewer**: So you have spoken of a number of a number of challenges, ahmm… from your point of view, what would be done to improve these services or to overcome these challenges?

**Respondent**: Yes, ahmm..we should not rely solely on the first day orientation sessions which really we know do not have the turn up of the students who have been enrolled in the university. There should ahmm… routine engagements with the students themselves to make them aware of some of the services that are being offered. And I believe even when we are having academic programs, there should be a responsibility of the lecturers themselves...they are informed that during the lecture, you can try and hint on some of these challenges these students face. For example in your class, you can use an example that makes students aware if the offerred services, even investing in extracurricular activities except academics. It is easier for us to get really consumed and focus solely on books when there are other thing that really help us have a holistic experience in the university and also build coping mechanisms that can help us when we do get mental health challenges. This includes sports, ahmm… engagements with different faculties, with different students, music, ahmm..prayer, you know, so all those different things have a role to play. Even the university can actually even involve ahmm.. let’s say the chaplaincy for the Catholics in their mental health problems whereby they work closely with them. I have seen universities where the chaplains are part of the staff and even paid because they are offering huge support to the student, thereby working with these Christian unions, religious bodies, sports arenas, all do different things to try and make an intricate mental health campaign. So it is not enough to just sensitize, there should be infrastructural investments. Then also, there needs to be an office, yes, like I said, the Dean of Students office deals with all those issues but it doesn’t suffice to the institution. I know that in the plan of the university there’s going to be all that in Kihumuro but even still some of these things are not cast in stone, it is like an idea that is being ahmm.. thought of but if at all, lets say, the buildings are placed, who knows if that room will be offered. There should be a help desk, a counselling room or office where students know that I can at least go there and get the help that I need. So I believe there should be infrastructural investment in all this in all aspects not just the name mental health services but finding out ehat other really ahmm…things in the student’s life can improve the quality of life they lead, can make them better able to cope with different things. That is what I really believe.

**Interviewer**: Okay. Thank you for those suggestions. Ahmm… Are you aware of any way in which these services that are offered are evaluated?

**Respondent**: Hmm… Ahh…I must tell you that the only way that I am aware they are evaluated is this: There is a student’s welfare committee and basically in that committee, the dean of students makes a presentation of what has transpired, what has happened in his office and then he makes propositions and then they’re dealt with. And then there’s ahmm…That’s what really comes to my mind. So the only way some of these things come to light, is when there are complaints made by students or student leaders, a gap being seen but otherwise this is not really one of the key things that the university is evaluated on. Ther’s more investment in maybe a graduation number, the passing rate, really it is more, there’s more investment in the academic sector than in the mental health sector, I must say. It is not one of those key aspects the university is evaluated on. That’s why I said it almost feels like some of these things are just put there to put a tick like tick Counsellor check, but do they evaluate his work? No, they don’t.

**Interviewer**: Something maybe I should have asked earlier is the services you used. Would you recommend them to someone else?

**Respondent**: I would. I would.

**Interviewer**: Has anyone else recommended to you a mental health service? Or have they given you negative feedback on the mental health service they got from the university?

**Respondent**: Ahmm…students especially from medicine, usually, recommend, well not necessarily recommend but they tell you about the psychiatry ward but there’s also, from what I have heard, even when you go to the psychiatry ward in the hospital, people who have been there don’t really feel welcome, they don’t really feel like they’ll get the help they need or you’re treated more of like a either you are ignored or you’ll be treated like an extreme mental health case. So it’s really a hit and miss at this rate, there are people who have had good experiences there are those that have had bad experiences. Can they be improved? Yes, they can. Is there a way of getting a response of people that have used these services and used them for evaluation? No, there’s not. What I would suggest is having a way of people reviewing the experiences they have had and then with an open mind, looking at these reviews and trying to improve where possible.

**Interviewer**: Okay. Thank you very much for your time. I just want to find out whether you have any further recommendations as regards mental health services utilization?

**Respondent**: Yes. Ahmm…there’s a gap in in…several in every other faculty including the Faculty of Medicine. But that gap is even wider in other faculties that are not directly taught about mental health services. So, even while there are challenges in faculty of medicine, imagine what is happening in faculty of applied science and technology. It is definitely much worse. There should be a breakdown of these services in all these different places. You find that even with the gap, it is much easier for a student in faculty of medicine than there’s for someone in faculty of applied science and technology will. We should be more aware in some of the differences in experiences and access to accessing health. We should not just make generic systems that basically rely on the person being aware of it and going to access the help themselves. The system should be tailor-made for different students of different years of different faculties of different lifestyles as well. There should be an involvement of the student themselves in some of these issues because like I said, it is the student around you that is going to identify that you have a problem. So, students should be made aware of these services because they are the ones that are going to be the first aid for the person next to them. It won’t be the counsellor to notify someone that they are ill So there should be an active and an almost equal participatory role with students and university infrastructure itself. Then, ahmm… the university definitely should ahmm.. re-evaluate the memorandum of understanding it has with the referral hospital, to really ascertain ahmm…what kind of help are we entitled to. I know for a fact that it is almost just like assistance the hospital gives and there’s no medical insurance not for any employee of the university, not for any student. Of course, this comes down to resources and I don’t want to justify why but there should more investment in making some of these services easier to access. So those were my suggestions.

**Interviewer**: Thank you very much. That is it.
