# Supplementary material for: Perspectives on mental health services for medical students at a Ugandan medical school
Source: BMC Med Educ. 2022 Oct 25;22:734. doi: 10.1186/s12909-022-03815-8 (PMC9592876; doi:10.1186/s12909-022-03815-8)
Supplement: Supplementary file 2 — Additional file 2. [file 12909_2022_3815_MOESM2_ESM.zip › Interview 3.docx]

**INTERVIEW WITH ASSISTANT DEAN OF STUDENTS**

**Interviewer:** Tell us about the mental health situation and services at the university basing on your experience in your years of service.

**Respondent:** The mental health situation at MUST generally has been good. I think we have been having reasonably stable students if I can speak on behalf of the student staff. But of recent, in the last two to three years, we’ve had an increasing number of students even before the Covid 19 pandemic getting admitted on the psychiatry ward with major breakdowns. The numbers have not been so alarming. However, when they tell you that in a semester 5 students have been admitted when previously you would get like one in the whole year it’s a bit alarming and, not a good trend. If I can also add, we have had increased cases of drug and substance abuse which could be what is causing these numbers to rise. But also post Covid, we can say there has been a lot of anxiety, a lot of tension, fear, people are not sure if they are going to pass their papers. A case in point includes a student who said, they were not sure if they were going to cope with their program when the semester has been shortened. So for them they thought if the semester is 10 weeks instead of the usual 17 weeks, they would not manage to study in those short weeks. Those were some of the challenges we have seen of recent after the last lockdown.

**Interviewer:** Given that you have indicated that there is a surge in mental health cases among students, we would like to know what mental health services are being offered to these students to help them cope with this

**Respondent:** Fortunately for those in the faculty of medicine, they get quick attention unlike other students in other faculties. Usually you find that the faculty and staff know that the student is not well but even when they come to us, we work hand in hand with the psychiatrist who is just a phone call away to let them know we have a student who is having a challenge. We take them physically and they are attended to. We also have students who have been admitted elsewhere, maybe in Butabiika or Mulago, wherever and they come when they have been referred for continuous review and we link them with the psychiatry department. We also do counselling, I am a counsellor by training. We have other counsellors and we link them up for support but of course we would always prefer the student to have a link with the psychiatrist and the family so that we work hand in hand. When a student breaks down, we actually go to their file, check for the next of keen, call that person, and if possible encourage them to come to let them know what is happening and engage them as well.

**Interviewer:** Basically it’s counselling as well as clinical management, those are the services that are being offered? (Answered yes). You have said that you tend to refer to the parents of the student, does that mean that these services are customized to the person receiving them?

**Respondent:** Not really but we inform a parent to know. Maybe the student has a previous history or it’s the first time this is happening so like any other sick student, we inform the next of keen, who in most cases is a parent so that they can come in and support. You that in health care we want people that are close to us. We do it basically for that purpose so that the parent doesn’t feel left out.

**Interviewer:** You talked about quite a wide range of mental health services. How do students get to know about these services, what efforts have you put to publicize these services?

**Respondent:** (Giggles) uhhhmmm, we rely on orientation. We usually let students know about these services during the first year orientation but of course it is not enough. We agree it isn’t enough but we also know that when the academic programs have started, it is difficult to come in with another service so we keep engaging through the guild and minster of health and see where we can. But of course you also know students are complicated to deal with. Even when we invite you for some of these good programs many of you will not come but we keep on giving out information. It is now easier with online things so now you just do something and put it out there, whoever sees it they read it, and if they don’t, at least you have put out information. We cannot force people to read or to seek service.

**Interviewer:** You have mentioned that you are a counsellor and I am very sure that you have counseled quite a number of people in your service, I would like to know how often students come to utilize these services?

**Respondent:** Those who need the service actually come, and those who other people feel need the service are also referred and we make contact. For example if you meet a student who you feel can benefit from counselling or any other service you let us know. We shall do the follow up

**Interviewer:** That means that there are students who get to know about the services through their friends?

**Respondent:** Yes. They get to know. For example, we have peer educators. We let them know and they can come up to us and say ‘we have seen this person and we feel we cannot handle and are asking you to make contact’. Especially in the medicine faculty, we get students who get concerned about their classmate and actually come and report and we take it on from there. Many times we actually get a group of students, like you have come here. They come and say they have seen a student who needs to be put into care and we proceed from there.

**Interviewer:** Basing on these services, can you please share some of the barriers that are limiting these students from utilizing these services being offered by the university?

**Respondent:** I think the barriers could be the stigma attached around mental health. For example there is a student who came here and I told her to go to the psychiatry ward and I think she almost hated me for it. She actually didn’t go and came back here when she was kind of worse. When I asked her if she went, she said, ‘no I could not’. I actually forced her to go. Fortunately when she went she did not have a mental illness but the psychiatrist was able to make the appropriate referral for the right service that this student needed. So sometimes because of that stigma attached, students don’t access the service and yet it is key/important. For example if I think a student is depressed, I might need a psychiatrist to confirm that using their criteria. I may have my thoughts about it, but I need it confirmed so that we can see how to support. Usually in mental health it is important to know the diagnosis you are working with. I know you have rotated in psychiatry and know that a person with depression is not managed the same way as a person with psychosis/anxiety, you need the diagnosis to plan for the appropriate intervention. But the moment you say that, the person says you think they are mad which is not the case. Therefore we need to demystify mental illness so that when you say psychiatry ward, people don’t look at the person naked on the street but know that it’s a lot more than that.

**Interviewer:** So stigma is a very big barrier. Are there campaigns or something you have tried to do to try and demystify like you have proposed?

**Respondent:** Of course, we need to start from demystifying counselling itself, because counselling is for many other things not only mental health. We need to start from there. How do we make it accessible? Because people will come when they are already hitting a wall yet they could have been helped earlier. But these are things we cannot force because you don’t know. If I meet you walking I will not know that you are unwell unless you tell me.

**Interviewer:** You talked about referring students to the psychiatry ward, and we know we know it is the only one in the whole of south western Uganda and has a very large catchment area. We would like to know if these students receive a special kind of care since they are from the university and the psychiatry ward ideally belongs to MUST.

**Respondent:** The psychiatry ward belongs to the hospital. (Laughs) fortunately because the psychiatrists are staff of the university, that works in our advantage. They have a number of people studying psychiatry here and that has been helpful. So you only need to make a call and the student will be received and given the right service. When they are not many, they have a small admission wing for students but if it is full, they get admitted on the general ward. They try as much as possible to give treatment to students.

**Interviewer:** Mental health in this time of a surge, do you think it should become a priority in this kind of setting where people are having post Covid era effects?

**Respondent:** Yes, it should be made priority so that people know they can be helped. Because during this lockdown, I think people have had more challenges and losses in terms of getting sick, we have had more people, staff and your fellow students getting Covid. Maybe even students losing loved ones to Covid and other conditions. I feel the stress level is probably higher now than the previous lockdown. It is like a carryover of issues from the previous lockdown. Now people are experiencing more losses and are getting sicker than before. I think mental health is something to seriously look into as an institution and as a country, it goes beyond the university as you can see.

**Interviewer:** Given that you have stressed the relevance of mental health, how do you think we could improve these services like in the delivery, making them readily available and known to the students so that they know they can come and utilize these services? Also share with us what your recommendations about it

**Respondent:** Of course the services have been there. I don’t know how else you want us to make them known because they are there. We are not saying we have reached the level of being overwhelmed. If people can come for the service, we are there to help. I am not saying we are many, I think the university has 4 people trained as counsellors but also other psychologists in the faculty of science. We can’t say we have failed, though the services are not being accessed. So how best we can improve the services is basically for your research to find out from maybe people, what they think. For as me who offers the service, I am there. It is a known service, like I said when we do orientation for first year students are thinking about other things that are going on in their minds and say they have never seen you. You realize they are not there mentally when you are doing orientation. I really do not know how best we can improve the information.

**Interviewer:** Even the services themselves, do you know how we can improve them?

**Respondent:** How? If a service is there, it is there. Do you want us to keep putting our pictures on noticeboards? I really don’t know.

**Interviewer:** If I may ask, if someone gets a service, is there a way of evaluating the service so they ensure constant improvement. Because we know feedback from that person could be important in improving the service

**Respondent:** That is now a tricky one. Help me understand it better. Are you saying if you came for a service, you would want me to ask you to evaluate the service?

**Interviewer:** No, is there a system where I could evaluate a service and the feedback from us who have utilized the service could help make it better for others

**Respondent:** We actually get the feedback. We don’t document it all the time appropriately. That could be the challenge. If someone has been here and I meet them on the walk way and we chat for two minutes and they say they are okay and they look okay, it ends there. Bu that is feedback. I think we are not a feedback giving population. It’s like when you treat a patient, they don’t come back to say doctor I am fine but they come back when they are not okay.

**Interviewer:** Some systems have put up models for example in some hospitals they assess and you can evens core the doctor basing on the care. In that line we are also asking is there a sort of follow up

**Respondent:** Yes we actually do follow up on some students. However when you are doing other things, you might remember to call when days have passed. In some instances we call to find out how students are doing but sometimes other things overtake.

**Interviewer:** Thank you very much. As we conclude, we would like to know your recommendations basing on your experience in this arena of mental health and services as one who has offered services to students

**Respondent:** What i would recommend is we need to demystify mental illness. If it can be demystified and we look at it as another form of wellness. You cannot be completely well unless your mental health is also well. We need to work hard on this to know that it’s okay to seek these services and help of these mental health professionals so that your life is better. In my experience I’ve noticed that if the mental health isn’t okay, the physical health is not okay as well. They kind of reinforce each other. We need to work a long way, all of us, you the students and the doctors. So that you don’t feel that if you find me at the psychiatry ward I’m mad. We need to get away from that.

**Interviewer:** Thank you very much for the interview. We shall utilize this information and we look forward to giving a report
