# Supplementary material for: Perspectives on mental health services for medical students at a Ugandan medical school
Source: BMC Med Educ. 2022 Oct 25;22:734. doi: 10.1186/s12909-022-03815-8 (PMC9592876; doi:10.1186/s12909-022-03815-8)
Supplement: Supplementary file 2 — Additional file 2. [file 12909_2022_3815_MOESM2_ESM.zip › interview 2.docx]

Key informant interview dean

**INTERVIEWER**: introduction and consent

**RESPONDENT**: there is no problem with the study, lets proceed

**INTERVIEWER**: thank you very much, as we start, we would like to know about your experience at the university... how have you handled the situation at the university? Tell us your role in the mental health situation at the university and how long have you been in this arena?

**RESPONDENT**: let me start by giving a background that my experience right from school has been working in institutions, in school… as a student, a pupil and after university education... after my bachelors I started teaching in a tertiary institution and I was still more keen on student welfare because I was a director of students then and then I proceeded through different institutions until I came to Mbarara university 20 years ago as a dean of students. so I have interacted especially with young students extensively for over twenty years and along the way I have interacted with them on a number of health issues and mental health has been among those that has been given less attention because its manifestations do not come out obviously in many people like many other ailments and many times by the time you get clinical manifestations then obviously then possibly the person who is challenged has goon through many other unseen stages … so over the last 20 years in must we thought that possibly interaction through peer groups… peer services can be able to identify walls hence that’s why we started the must peer project way back in 2004 and the first time the main concern was to deal with sexual and reproductive health because the invisible challenge then was HIV aids and so we started the peer project… along the way we realized that peer services are important but we need psychosocial services to give the necessary support to students so we started counselling services and employed counsellors at the university to give psychosocial support to both the counsellors and the students. Of course, the counsellors were working with the clinicians in medical school and hospital at the psychiatry unit so that as we give supportive care, we give it with a background of clinical support for students and staff with clinical manifestations … so that’s how we have been managing over the years with basic services for care and support. But as the university grows in numbers, those challenged increase and it appears overwhelming……I hope that gives you a background and myself….

**INTERVIEWER**: thank you very much. that has been very informative… you have talked about students getting counselling and students also having support groups which will help them cope with mental illness and mental health... you have pointed out that the mental health of students is not given as much attention given that its manifestations are not as severe as those of physical health… we would like to know if students utilize these services as much as you expect from your office…

**RESPONDENT**: globally mental health services have not achieved the rightful position in health care services… usually there are few students who really feel stressed and feel they are getting overwhelmed so they come walking in looking for somebody to talk too… they are looking for somebody to talk to not because they are sick but because they feel they have a challenge with which they feel they need support and that is usually the beginning of the care we give…some of these students will come with issues to do with academics .. sharing with us how they are challenged and getting difficulty to cope with their studies. Some students will come with issues to do with their background in terms of their health where they could have had a mental breakdown. Some will come to share challenges of how they are finding difficulty to relate with their colleagues… some come and talk about their family backgrounds …etc. etc.

But we also get their concerned colleagues who will come and tell us … “we think of late our colleague has been appearing in an unusual state… they are available but miss lectures, they aren’t attentive… they are behaving in ways that require close observation…” that’s where peer support comes in that sometimes a mentally challenged person may not be able to realize on his or her own but may need care team. a care support of concerned family members, friends, peers… to come and say look something has changed… whats going on… that’s where peer services come in and at that level that’s when we look at the professional bit of support … in terms of professional support, clinical support, counsellors.. if need be, we look for clinical care … based on how we think the counsellors are advising and some times we get information in the records, family members, parents or guardians

This leads us to the referral system from ordinary discussion from trying to help a person cope with a personal problem moving on to professional counselling from someone trying to see how this person can be brought on board progressively to clinical psychiatrists who may tell if the person needs to be assessed and maybe initiated on treatment then to clinicians who may give the care to people who may need to be regular attention or even in extreme cases who may need admission... that’s the kind of structure that we have at MUST

**INTERVIEWER**: thank you very much sir… I would like to know how many counsellors does the university avail to students

**RESPONDENT**: currently we have two full time professional counsellors that are provided by the university in the department of dean of students but also have other counsellors under the faculty of medicine who work with the psychiatry unit and support the same group so all in all we have at least 5 counsellors with whom we laisse with as a department

**INTERVIEWER**: are the services offered by the university publicized. in otherward how do the students get to know the services that are available and can be utilized at any time they want?

**RESPONDENT**: the starting point is during the student’s orientation, during the orientation week when students report for first year, that’s when we introduce them to the various services that are available…. we do realize that we don’t capture the biggest percentage during orientation because a number of students report late and possibly some of them are a little diverted. On the other hand we also have student leaders whom each time we get new student leaders through the guild elections … we also have regular meetings with them, we invite the new leaders… the grc… the guild executive and some leaders of associations ... we also usually bring out these information to them in the hope that they are able to communicate to their constituents … so of course the other area that are not very much accessed is that information is available on the university website if you go to the link that shows you what the dean of student’s department at MUST does. Of course, I realize that not many students use those links... I also want to imagine that the student guild on their link should also be able to have that. I also didn’t possibly highlight that we have university clinics… we have university clinics at the town campus and we have also got one at kihumuro campus… so we also have got some nurses stationed at those clinics who also sometimes also receive those cases that they report for onward referral.

**INTERVIEWER**: ok… so. thank you very much…you have talked about ways through which these services are publicized to the students … I would like to know what barriers have you, your offices and the counsellors attached to your offices have faced… what barriers have they faced in availing these services to the students….

**RESPONDENT**s: the staff generally are available however it has also been noted that the perception of many members of the community… they tend to think that if somebody needs mental health services… then literally the person is either mad or running mad… and therefore there is still some stigma associated with referring clients. So, when for instance a student comes and they are explaining what they are going through and u realize that it is not necessarily a casual kind of temporary challenge and you suggest that “do you mind I make an appointment for you with a professional counsellor...?” some will gladly say yes while some will say. I think there is no need I will be ok … now when they go to a professional counsellor. those that give psychosocial support and they say “we think you should talk to a professional psychiatrist…” that is where usually the tag of war begins. the students will say “psychiatrist… no… but am not mad …” many times with support of fellow students… with support with the university security teams we have to actually force them to work with the psychiatry unit to actually handle some students and force them into psychiatry unit for care … so that kind of stigma that initially not many people will voluntarily walk in the psychiatry unit for care because of the thinking mental health is associated with madness which is not necessarily the case at all because the biggest part of the population at one point or the other will need that kind of psycho social support or even clinical support for mental related ailments so the biggest challenge has been the clients reporting and also voluntarily accepting to be referred depending upon their level of ailment.

**INTERVIEWER**: ok. thank you very much sir… so. you have talked about utilizing the security and also maybe use of force sometimes to overcome these barriers. what other solutions have you looked at to ensure that you overcome these barriers such as stigma to utilize the mental health services by the students and also the reluctancy of students to approach you or the people offering this services because you highlighted that at one point these students don’t attend orientation when you offer the publicity for these services and some even don’t access the links to the university website where you also publicize the services… so which solutions have you come up with to help and handle this situation?

**RESPONDENT**: well, I think… (informant gets a distraction).

After 15 minutes …

**INTERVIEWER**: our informant is back so I think we could proceed... we had asked about how best we could improve these services for the students to have a better experience while accessing them and utilizing them? thank you

**RESPONDENT**: now I was saying that we need to mainstream mental health issues as one of the health care packages we have at the university… as the population grows we expect to get people with challenges from the different backgrounds and therefore we need to make sure that our system can easily cope with the emerging challenges because it is now eminent that we get more and more students who are actually experiencing different forms of mental health exacerbated by a number of environmental factors such as unregulated alcohol use, increased penetration of substance abuse and many other challenges that we get exposed to through the various environmental stressors wo we need to mainstream it and also look further and see why do we seem to be experiencing more reported cases of mental health… what are the drivers that we need to address as well… instead of waiting for the symptomatic cases…we should also look back and find out what could be the causes of the reported increased concerns to do with mental health so we should not look at it exclusively from the clinical set up.. we should look at the environmental issues as well as background factors that manifest because a number of the people who come up with mental health challenges don’t necessarily pick them from campus… some of them have a history when you talk to their friends and relatives… for those with a history … how do we capture it… for those where it gets worse at the campus… what makes it worse… so we need to look at the issue more broadly beyond institutional support and factors beyond the institution…

**INTERVIEWER**: thank you… I would like to find out if there is a mechanism that is in place to evaluate these services that are offered… the counselling… the social support or group therapy… the psychiatry… you know… clinical support…is there a way or a form of evaluation to ensure these services are updates and tailored to the student needs or the students who seek them up...

**RESPONDENT**: currently we have not had any evaluation am aware of and maybe I think what you are raising is something that needs to be addressed holistically to find out our level of sufficiency and preparedness so we do not have an evaluation that has been done to the best of my knowledge…

**INTERVIEWER**: you have talked about how we could improve… how we could evaluate barriers to the services that are being offered… it has been an informative talk but then I would like to find out if at all there is a scenario you have had in your years of service at the university that touched your heart in regards to mental health and mental health services that you could share… what do you think you would have done better... what were the positives that maybe you would wish to share from such a scenario.

**RESPONDENT**: well, the … I have really experienced many cases but probably the two related cases I am looking at is where at the end of the day people associate mental health with witchcraft and then you look at this student who needs clinical support and some of them initiate treatment and get better… when you get back their care givers especially back at home... instead of supporting this student to adhere to medication… they insist this is witchcraft… withdraw the patient from the campus and hospital and take the patient back home … often, the patients get worse because when the go to the society they start looking at the family history .. so maybe they say … “even another relative had mental issues…” so one unfortunate incident was of such a student who was retrieved from the university and hospital and taken back to his home area and when we were making a follow up from the friends and relatives they told us that the person had died because I think along the way he contracted other ailments and I think got other sicknesses perpetuated by the mental ill health and people kept thinking that it was the mental ill health until when the student died… that was 10 years ago… and it was very unfortunate that somebody whom we were looking at as responding to treatment is actually withdrawn from care and unfortunately succumbs..

**INTERVIEWER**: thank you very much sir… as we conclude I would like to find out from you if you have any recommendations following your experience at the university towards mental health and mental health services in general… what recommendations would you make to increase student accessibility to services as well as increase effectiveness and efficiency of these services to people they are intended to serve.

**RESPONDENT**: first of all, my main recommendation would be that we expand our support services to include information about drug substance abuse so that we know that… because apparently from the hospital records many of the students who report with mental health issues have got some bit of background of substance abuse. And then two. also, to indicate that counselling services are ordinary services that we consume because of the stresses within our environments and not necessarily because we think we are running mad. 3. To know that mental health is part of the overall requirement of the human being like any other health concern that somebody who has reported having mental health should not be stigmatized because we look at individuals health holistically not only from what we look at as physical fitness so that at least we have a community that is very conscious about our mental health and also a community that is very supportive and to say that actually mental ill health is a medical condition like any other and we should not necessarily attribute it to superstitions and witch craft so that when we main stream this that even somebody who has been under clinical care will confidently walk into the clinic for support.. will confidently go in for review and if somebody needs additional support then a person can say that for certain reasons this situation may not be suitable for me. it might be a stressor… so that even when students report. students can say with this environment I am prone to a breakdown. and also know that there is a patten… it tends to occur largely under… if there is a situation of increased stress. towards exam period. towards the pressures of paying tuition so there are also other pressures but if they are communicated to members of faculty then we can see how we can be able to cope with some of those individual variations when we are organizing certain actives and programs and of course also when you are deploying like say in case members of staff… when you are giving assignments and when you are deploying you bare in mind that this environment. this situation might add to somebody’s stress so that the more we open up as a community. the kore we open up as individuals. the more we are able to find timely solutions… thank you

**INTERVIEWER**: thank you very much sir for the interview; we surely are going to use this information to probably make a good study that could be used as a reference in policy making in your office and higher offices. We thank you for accepting to participate in our study

**RESPONDENT**: alright … I look forward to your report

**INTERVIEWER**: definitely we shall share it

**RESPONDENT**: alright
