# Supplementary material for: Perspectives on mental health services for medical students at a Ugandan medical school
Source: BMC Med Educ. 2022 Oct 25;22:734. doi: 10.1186/s12909-022-03815-8 (PMC9592876; doi:10.1186/s12909-022-03815-8)
Supplement: Supplementary file 2 — Additional file 2. [file 12909_2022_3815_MOESM2_ESM.zip › interview 1.docx]

**Interviewer:** So we are going to record the interview, if you don’t mind. Hope am audible.

So what, so now this one is to know about you, we shall not take your name, we see your female, what do you around this place basically?

**Respondent:** Aaaargh, well, am an SHO, so study and treat patients, the mentally ill patients and also to teach undergrads.

**Interviewer;** Thank you, so how long have you been here?

**Respondent;** Aaargh; since January, so I think, since December so I think eight months now.

**Interviewer;** Do you feel that you interface with the students abit often and talk to them maybe about their mental health (and all that)?

**Respondent;** Aaaargh no, only when they are presenting (smiling) cases is when I interface with them.

**Interviewer;** (interface with them……) so do you know about any mental health services that are provided by the university to the students?

**Respondent;** Apart from education and and (smiling) treatment for those that are ill and come for the treatment, that’s as far as I know.

**Interviewer:** So, you have not seen any yet? Aaaargh Like students or people that are coming from the university that are……

**Respondent**; I have seen, about one or two that are sick or around three, they are just there up to, they come when they are having mental illness not when they are………… maybe just having some, aaaammmh maybe……… you know mental illness is on a scale from good to bad… so aaaaammhh everyone everyday goes through aaaaargh variations of mental illness. So, its not that they come when they are going through something like in the prodromal period before like they actually break down, no, they come when they are sick, when they need medication.

**Interviewer;** so, which…...which common kind of mental health issues have you seen students presenting with here?

**Respondent;** aaargh bipolar, depression, adjustment disorder, aaand schizophrenia. Yah.

**Interviewer;** So, these students, do they usually come on their own or they brought by friends? Do they talk about how they found out they can access this service?

**Respondent;** Only one has been…has come on their own, by themselves. The rest are brought by students or by friends or by parents.

**Interviewer;** so, do feel like the stu… the treatment that you give the students when they come under the umbrella of the university is customized to those particular students or you treat them like how you treat the normal patients is there any special …….?

**Respondent;** Yah… they are actually given special treatment. Usually the seniors don’t want them if they are admitted, they want them in a private setting in the private room no on the general wards like everyone else unless the rooms are filled up and they are usually given more attention the seniors keep, you know following up and checking on them and actually there was one student, you know the drugs available here are typical antipsychotics which have many side effects. So, and apparently atypical antipsychotics are better so aaargh they…. I’ve heard of a case where aaammmh is it the dean or the medical director I don’t know aargh calls to ask which treatment the patients need so that the university can get them the treatment. So they are actually treated in a very special way.

**Interviewer;** so... (clears voice) do you think that….do you feel like the services are very satisfactory hoe they provide it to the students and do they feel free to always come and consult in case of maybe their medication is sort of giving them trouble? How do you usually go about that?

**Respondent;** aaargh i…….do not think that students feel free for some reason, it could be due to the stigma around mental illness. Aaargh because I mean sometimes stigma is not what people, how people treat you, sometimes stigma is what you feel.

**Interviewer;** you feel and think about it yourself.

**Respondent;** yah so think its about. Around the stigma most not…. I mean I’ve seen few for the period I’ve been here or they have been admitted except on with bipolar who wanted his medication changed because of his. Of the adverse effects of the drugs but he wasn’t comfortable. He…even whenever he comes its not that he can talk to any of you. He he he’s… just looks uncomfortable. If you find him on the streets, he doesn’t want you to see him, something like that. (interviewer; ooooh) yah so I don’t think they are comfortable at all. Even when you meet them in the university as you’re walking, and they know you’ve admitted them on the psychiatry ward, you’ve treated them, they avoid you or they pretend like they did not see you. So… I don’t think they are free.

**Interviewer;** they don’t want people associating them with you in that sort of way…...

**Respondent;** yes…yah that kind of associated stigma, its like how aaargh I think it comes to even when you’re doing psychiatry, people will think you know, you’re like your patients or something like that. So I think it’s the stigma around mental illness.

**Interviewer;** okay…...do you think there any other factors that could hinder them from coming here to access services and maybe going through the care as it should be?

**Respondent;** well personally my opinion aaargh I think either the university or the hospital has a role to play, because mental illnesses are part of us but aaahhmm………no one seems to acknowledge that we have three psychiatrists in this department who are also very busy and they are doing research, they are doing there…. they are teaching us, they are teaching you know the undergrads. And there I heard about fifteen pediatricians and mental illness is going to happen in everyone no just the people….

**Interviewer;** in every department………

**Respondent;** yes, in the other departments, there are people, as long as you fall sick ideally, its not…. your mental illness is affected your sorry your mental health is affected. But I don’t think its given priority at all……. it is looked at like, so I think it has a role to play………with why because if the university or the hospital aaargh acted like it is you know a normal thing we are all going through it, we are all mmmh …. These are the psychiatrists; these are…. you know the residents. There is special attention given to this area because it really affects the rest of your functioning.

**Interviewer;** so if you are saying that we do…..we….. there. There is…the attention is not enough. Do you think it would be better if they sort of introduced the psychiatrists? How do you feel like that we can solve that problem?

**Respondent;** exactly exactly…...if maybe you know they introduced you know right from maybe year one, these are the psychiatrists, these are mean know, the residents course the residents keep changing. These are the couns….the psycho….. this is the psychologist, the social worker, I mean the whole hospital university have one psychologist eeeh and for every patient, you will need a psychologist. The burden is too much on them, they can’t, it’s not about…. if…. What if you just need you know counselling, what if you may need cognitive behavioral therapy, what if and that is the one person that has to treat everyone. So, I think it also comes down to that, the attention, there is no attention given to the psychiatry department, to mental health in both the university and the hospital, mean there is one social worker and one psychologist, so…...

**Interviewer;** so, when these students come to you like the ones you said have been admitted here, do they have access to all those people despite the fact that we have one psychiatrist and one social worker.

**Respondent;** Unfortunately, not…...

**Interviewer;** it’s not a multidisciplinary thing?

**Respondent;** I don’t think any of them has seen a psychologist, that’s the truth, I don’t think so.

**Interviewer;** So, they usually just see a psychiatrist, get medication……...

**Respondent;** Yah, the medication and then you give them the counselling that you can give, you give them the best you can do but the other person is better trained in that area.

**Interviewer;** so, as a psychiatrist you also get training to do some sort of counselling?

**Respondent**; yes, it’s part of aaaargh it’s like how you are doing medicine and you do some bit of radiology, some bit you know all those things, some you go to the lab, some bit of pathology, does not make you a pathologist. if you venture in that area, if you concentrate, yes, or if a lot of your attention is there, you will but then you are needed here also………………... more than……. yah

**Interviewer;** so, what are some of the other things that you think the system would improve such that students are able to know about these services and come and seek help in case they need them it because from what you’ve said it was only one student who brought themselves, the others were brought by friends or teachers. So how do you think that as a university can improve to find that students actually come and utilize and know that these services are present here.

**Respondent;** ooooh aaargh (exhales), as a university, I I………. don’t think psychiatry should just be like a course unit you do in fourth year. I think its there in fourth year

**Interviewer;** yah the year where we basically, our minds have switched off, we just pass through the system (smiling).

**Respondent;** yes, I feel like these are things that so many people don’t even know they are mentally ill, even medical students don’t know they are mentally ill, even when I practiced as a general doctor, I got patients and I didn’t know they were mentally ill. It now that am specializing am realizing, wait the other patient actually had this condition. So, I feel like because it happens a lot, it is with us, I feel like it should be introduced right from year one not just as a course unit but as some sort of advocacy, some sort of you know education once in a while, like aaaah I think there are……I think there aaammmh …..how can I call it? Like how people have like some sort of organiz…… I don’t how they call them here. (interviewer; like discussions or clubs? Yes we do)

Aaanha something like that where aaargh people are just, you know educated about this briefly, these these are symptoms of early mental illness. If you’re experiencing these, please go see a psychiatrist before it gets to the others…. You know, if you’re…... things like that. I feel like it should….

**Interviewer;** Who do you think is most equipped to come and take part of those discussions because in the university we have some of those and its usually the students they look for an outside facilitator. So which better way do you think it cause……...

**Respondent;** I think it’s the people here. Because if it’s the people in this department that are talking to you, you you will know that there is doctor X, there is doctor Y she has talked to us other than getting an outsider that you are not going to see.

**Interviewer;** hmmmmmh

**Respondent;** hmmmh, when you break down, you are going to come to the psychiatry department.

**Interviewer;** and I think it helps if I have seen them before...

**Respondent;** if it’s a familiar face. Hmmmh, I mean we interact with people and and and you say okay I think I will feel more comfortable with this one or the other one or... So when you come to the department, you’re looking for someone you know, something like that.

**Interviewer;** so from our discussion like I told you we looking to see which mental health services we have and you’ve said you’ve only seen people who have come here to get admitted so you don’t know if we have counselling or some sort of activities that kind of keep encouraging the students about their mental health. Have you heard of any?

**Respondent;** I don’t know about them, no...

**Interviewer;** not even in the corridors that you heard? (laughing…)

**Respondent;** No, I haven’t.

**Interviewer;** you haven’t heard any…. okay.

**Interviewer;** So for you, so aaargh which other thing do you think as that could help as a mental health service like what is the thing that we could do to tease out people that probably need the mental health service because even in discussions most times as you realize campuser really do not like going in to sit and listen to discussions. So how do you think you can entice us more to realize that we have the problem and need to seek help.

**Respondent;** (smiling) well I get what what you’re saying there, I I how many would want to go and sit to a ……….. (I didn’t this part clearly, (12:00-12:30)

**Interviewer;** hmmmmmh Yes, people will see me and say ooooh she has gone, she probably is mentally ill. So ….

**Respondent;** Oooh, yah stigma, I honestly, I will repeat the same thing I said it should starts from the top, if the top people recognize that… you know this is an important area, this is an important and they, aaargh talk to the students like its….aaargh I will give an example, aaargh what was it? if you want people to use aaargh family planning for example, you don’t… you have to……you you it starts from the top, its.. if the government is telling people, advertising family planning services, immunization services, people out ther will realize its necessary but if it starts from down, someone comes up and says oooh this is important. Hmmmmh. A few people may believe, some won’t believe so it should start from the top, the the manager, the people running the hospital, the people running the university are the ones who are first supposed to recognize that this is important. And find ways of addressing it to the students. If the students are hearing it like this is routine, this is normal, they will take part, that’s what I think.

**Interviewer;** hmmmmh

**Respondent;** I don’t think it should just come.. if a student just comes up and …. you would have to be very interesting or a celebrity of sort. Very……. You know, but it should come from the top.

**Interviewer;** so the university should find a way of emphasizing it from an early stage and not just…….

**Respondent;** Yes, from the time you join university.

**Interviewer;** and not just, cause right now its only the medicine class that gets to hear about it.

**Respondent;** Yes, it should be everyone.

**Interviewer;** so aaargh doctor you talked about aaaargh youth.. you talked interfacing with students here on ward, do you think aaargh there are….what facili what are the facilitators you think are helping some of these student to come apart from lemmi say their friends holding them and also them knowing about the service?

**Respondent;** I’ll tell you, what is..aaargh… I think its just their friends. No.. Because usually, even, I’ve seen cases where their friends bring them and they tell you aaargh this person has been like this for about three weeks, so they bring them when now they are becoming psychotic, they are starting to see snakes or you understand? They bring them when its….

**Interviewer;** So doctor, also you said stigma is a very big barrier towards accessing these mental health services. What do you think has been done to help? What other barriers do you think exist towards lemmi say people knowing that there is a psychiatry department and as a university student I can go there and I receive care? So what other barriers do you think exist towards people accessing the…..

**Respondent;** Not knowing that there, besides stigma, they are not told that these services are available.

**Interviewer;** So right now from our discussion aaargh we know that the service.. the psychiatry department is one of the mental health services that are being offered to the students, you’ve said that students who come here receives special care which is not what other people receive and the university sometimes helps to foot the bills. Now what has been done, because you said barriers are people not knowing, do you think something has been done to to solve this hurde?

**Respondent;** As far as I know, No, maybe something has been done and I don’t know but I don’t think so.

**Interviewer;** Alright, so when you treat these students aaargh here at the psychiatry department, aaargh one do you think these students tend to appreciate the services that are being offered to them?

**Respondent;** I have never bothered to ask, I wouldn’t know if they appreciate or not.

**Interviewer;** So there is no like form of evaluation? Is there follow up, files that are for students. Cause we know that we usually have files, do they keep the ones for students on the other side and are followed up to come and see if they come and get their medication?

**Respondent;** No, they are not, the files are not kept in any separate place, they are all kept together but I mean everyone who is discharged is given a review date and we always probably just assume that these students have been here, they they know when to come back, something like that.

**Interviewer;** so do you think, aaargh, because you’ve…..amidst the stigma and the special treatment, you know, do you think these medical students would recommend other friends of there to come and use the services being offered here?

**Respondent;** Aaaargh i’ll say this, if you’ve…. If you’ve…aaargh none of you has been in labour (laughing). If you’ve had a surgery somewhere, usually by the time, like I said most times they come when its really bad, if you’ve had a surgery, and it was an emergency surgery. Maybe you’ve had this appendicitis and then you go when it’s you know, ruptured and…and you’re treated in this hospital, and you’re treated well, I think it will depends on your knowledge to tell or on your interpretation of things hmmmmh to tell someone else that don’t wait for this stage, go here or when if finds someone else in a lot of pain then you’ll tell them go to this hospital hmmmh but if you find someone with mild pain, you may actually also tell them to just get some painkillers or to just get over it or go and…. you understand? So I think it…… it will go from someone’s, it starts from someone’s understanding and.. and knowledge of things. If you came and I treated you here because you were psychotic hmmmmh, you were not even understanding what you were doing so I treat you, you get well, you go back. Sometimes its…. its hard to differentiate other people’s personalities from psychotic features, some people are naturally paranoid for example, some people naturally believe….so you may not know that this is when I am supposed to advise someone to go to hospital.

**Interviewer;** so you’ve hinted on aaarrgh there is lack of awareness aaaargh in the general population especially medical students whom we are investigating about but then. I want to focus on the services themselves. Yah, you’ve told us that sometimes the faculty intervenes and they pay for the bills and everything but is there something, under your observation for the people, the patients you’ve interacted with and are medical students, are there things you would wish to improve?

**Respondent;** aaaaargh to improve about………

**Interviewer;** The way they are……..service delivery and everything,

**Respondent:** service delivery here?

**Interviewer:** that whole whole chain.

**Respondent;** I think that……… something that should be improved is maybe increasing on the number of psychologists and…. its about the staff. Because mental…. mentally ill patients, the admitted ones are quite hectic, hmmmmh, you’ll see one patient for three hours. Just one patient, hmmmmh, because you want to understand what’s happening, you need to counsel, you need to go to the roots, and after that you need to see another. Actually, ideally, they tell us that if you see four patients in a day, you’ve not seen them, you understand? You’ve not given them the best you should. So aaargh the staff are limited that’s a fact and that not something I can’t change as me. But also, I’ve also been wondering there seems to be, there is a high rate of aaaargh mental illness in in Mbarara University for some reason. I wasn’t here for my undergraduate, I was in Makerere but when I came here, its… its alarming I don’t know what is happening, so I don’t know.

**Interviewer;** That’s why we are doing this research. We want to know the route cause. So aaaargh probably we think it could also aaaargh be following the Covid-19 pandemic, a lot of people are breaking down and its alarming, that is very true, its alarming. And aaarrgh, we hope that results from from such studies can inform policy, can inform even the students themselves to start probably taking care of themselves aaaargh yah better.

So maybe as we conclude, are there aaaaargh mechanisms for evaluating the services you offer here, cause you said yes it’s on a personal level someone to recommend but do you think there are mechanisms that people can use?

Respondent; that are in place?

**Interviewer;** Yah that are in place or the ones you can recommend or when you talked about when students are admitted here, the follow up process is there any kind of evaluation to see that the end result is good and the students are benefiting? Because at least we know that these staffing here is under the university and not under the hospital. So is there a way of evaluating these services.

**Respondent;** I don’t think there is any in place. What we usually do, I think its general, to see improvement of our patients is there a relapse? How many relapses?

**Interviewer;** maybe the final one is in your practice; how how how long did you practice before coming back for your master?

**Respondent;** Well I graduated in 2018, January so before covid time

**Interviewer**; Are there any service, mental health services that have been provided that you think as a university here we could benefit from if we incorporated them for students?

**Respondent;** aaaargh, you need to make me understand the question.

**Interviewer**; okay so because we’ve talked about how you feel like we could inform students better, so among in you practice the people that you’ve met maybe people that are our agemates with mental illness, do you think there is some of the…… what various ways have they been managed that side that we can incorporate here?

**Respondent;** telepsychiatry…...

**Interviewer;** psyche…. the what

**Respondent;** Telepsychiatry is is like telecommunication like zoom. I personally actually have a YouTube channel where I advocate and teach about mental illness. So through that, through media, some people will be more comfortable hmmmh aaaargh communicating by those…..because of ……like even if you try to reduce the stigma, it’s like HIV, even if you try to reduce stigma around HIV, it may go down a bit but it won’t really, the stigma is still be there. So I I… It could be the same thing with mental illness I don’t know. No one has really tried to you know fight stigma on mental illness in Uganda, I don’t think anyone has but like, I feel like it’s about finding better ways of passing on this information to people or letting the students know that you can access you know, services through media. You don’t have to…. if maybe someone contacted me through media and and… maybe I need to meet them privately, that can be arranged than someone coming to…… So I think telepsychiatry is something that should be adopted. I mean we live in the covid, everything is going you know that direction so I think telepsychiatry helps.

**Interviewer;** Alright, thank you very much doctor for your insights and wonderful input. We very much hope that the results are going to be beneficial. And aaargh unless my colleagues have something to say I would like to end this……. thank you very much doctor.
